# Tsunami modelling over global oceans

**DOI:** 10.1098/rsos.241128

**Published:** 2025-01-15

**Authors:** Siva Srinivas Kolukula, P. L. N. Murty, T. Srinivasa Kumar, E. Pattabhi Ramarao, Ramana Murthy M. V

**Affiliations:** ^1^ Indian National Center for Ocean Information Services (INCOIS), Ministry of Earth Sciences (MoES), Hyderabad, India; ^2^ Ministry of Earth Sciences (MoES), Government of India, New Delhi, India; ^3^ India Meteorological Department (IMD), Ministry of Earth Sciences (MoES), New Delhi, India; ^4^ National Centre for Coastal Research (NCCR), Ministry of Earth Sciences (MoES), Chennai, India

**Keywords:** tsunami, modelling, ADCIRC, wave height, warnings, tide gauge

## Abstract

Tsunamis are massive waves generated by sudden water displacement on the ocean surface, causing devastation as they sweep across the coastlines, posing a global threat. The aftermath of the 2004 Indian Ocean tsunami led to the establishment of the Indian Tsunami Early Warning System (ITEWS). Predicting real-time tsunami heights and the resulting coastal inundation is crucial in ITEWS to safeguard the coastal communities. Global tsunamis other than those in the Indian Ocean might weaken at Indian coasts due to distance yet still cause significant damage due to local coastal morphological amplification. The current study focuses on tsunami simulations over global oceans. A finite element (FE)-based ADvanced CIRCulation (ADCIRC) model is configured to the global domain to model global tsunamis accurately and efficiently. The model mesh has a spatial resolution of 2 km in the shallow waters and relaxed to 20 km in the deeper waters. Model simulations are performed for significant historical events, assessing their effect on near and far field regions. Computed results are compared with the observations, and it is found that the model’s predictions align well with the observations. The simulation results demonstrate that ADCIRC can be applied to real-time tsunami predictions due to its computational efficiency and accuracy.

## 1. Introduction

Tsunamis are a series of enormous waves created by an underwater disturbance such as an earthquake, landslide, volcanic eruption, meteorite or atmospheric forcing (IOC/2008/TS/85 rev.4). In the annals of modern history, 26 December 2004, the Indian Ocean tsunami remains one of the worst natural disasters [1]. Triggered by a massive 9.1−9.3 moment magnitude earthquake near Sumatra, Indonesia, at 00 h 58 min 53 s UTC, it resulted in catastrophic waves, causing widespread devastation in numerous nations bordering the Indian Ocean, including India [2]. The generated tsunami killed 230 000 people and destroyed towns, villages, infrastructure, livelihoods, fisheries, tourism and the nation’s economies were crippled. Despite the challenges, it catalysed global cooperation, technological advancements and improved disaster preparedness, leading to a stronger and more resilient future for vulnerable coastal communities [3]. This event prompted the establishment of the Indian Tsunami Early Warning System (ITEWS) at the Indian National Centre for Ocean Information Services (INCOIS), an autonomous body under the Ministry of Earth Sciences (MoES) in October 2007 by the government of India. ITEWS integrates real-time earthquake monitoring, sea level observations and advanced numerical modelling and operates round the clock to predict potential tsunami threats to issue timely warnings across the Indian Ocean [4]. The primary objective of the Indian Tsunami Early Warning Centre (ITEWC) is to detect, locate and determine the magnitude of potential tsunamigenic earthquakes (>6.5 Mw) occurring in the Indian Ocean Basin and provide timely advisories (Warning/Alert/Watch) to the vulnerable community by following a certain standard operating procedure (SOP) ( [4]). The Arabian Sea and the Bay of Bengal are prone to powerful earthquakes due to Sumatra and the Makran subduction zones, which can directly trigger destructive tsunamis impacting Indian coasts.

Accurately predicting the arrival time of tsunamis and wave height at coastlines and inundated areas is critical for effective evacuation and mitigation efforts. Various advanced mathematical models are used to model and predict tsunami wave dynamics. The shallow water equations (SWE) obtained by integrating Navier–Stokes equations along the depth, assuming vertical acceleration is small compared to horizontal acceleration, capture the tsunami behaviour [5]. Depending on the availability of high-performance computing (HPC) facilities, various numerical methods are applied to solve these SWEs and simulate tsunamis. Numerical methods like finite difference (FD) [6–10], finite volume (FV) [11–14] and finite element (FE)( [15,16] and smoothed particle hydrodynamics [17–20]) are employed for simulating tsunamis. These numerical models need the initial sea level disturbance due to seabed displacement caused by the earthquake thrust. The fault parameters needed to calculate initial displacement are longitude, latitude, depth of the earthquake, length, width, strike angle, dip angle, rake angle and fault slip. Once these parameters are known, the Okada solution [21] can calculate the initial displacement or deformation. Tsunamis in real time are also forecasted based on the pre-computed scenario database. A pre-run scenario database results from numerous tsunami simulations for various potential earthquake scenarios or a model simulation result calculated with the initial seismic deformation condition against the pre-defined input fault geometry [22,23] across a specific region. These simulations pre-calculate and save tsunami wave travel times and run up for different earthquake magnitudes, locations and fault mechanisms. In real time, when a tsunamigenic earthquake occurs, the closest focal combination is picked and scaled to match the actual scale.

## 2. The objective of the study

ITEWS issues warnings based on pre-run database scenarios [3,4]. As a redundant option, ITEWS explored running a numerical model in real-time [24] based on the available HPC facilities once fault parameters are estimated. ITEWS INCOIS has demonstrated the capabilities of the ADvanced CIRCulation (ADCIRC) model, an SWE solver to compute tsunami wave heights, wave arrival times and inland inundation once the seismic fault parameters are known by launching the model in real-time [24]. ADCIRC is a fully parallel continuous Galerkin Finite Element based shallow water model that computes the water level and currents at different scales on an unstructured grid [25]. The model was set up for the Indian Ocean, which covers the potential tsunami threat from the Sumatra and Makran subduction zones. The current work aims to extend the ADCIRC model to set up for the distant global tsunamis. Distant tsunamis, also called teletsunamis, are a series of giant waves generated by large-magnitude earthquakes that travel thousands of kilometres and reach the global coasts, creating significant damage [26]. Teletsunamis can travel across different ocean basins for hours before their arrival, and local factors like coastal geography and underwater features can amplify the tsunami waves in specific areas, increasing their destructive power. Because of their long-distance travel, these global tsunamis give sufficient time for issuing warnings and evacuations. In our present study, we have utilized ADCIRC to simulate distant global tsunami propagation and its characteristics at various coastal regions over the globe. We have simulated a few significant historic global tsunamis and exhaustively compared the results with many observations to validate the current set-up. This study utilized records from about 180 tide gauges globally. This is the first-ever study that proposes real-time tsunami simulations on a global scale using the ADCIRC model and is one of the few studies to validate model results using 180 tide gauge records. The fully parallel ADCIRC model enables real-time simulation of global tsunamis, allowing ITEWS to pinpoint coastal hot spots and issue prompt warnings, potentially saving lives.

## 3. Methodology

The major steps involved in the numerical simulation of the tsunami are generation and propagation. Generation includes generating initial disturbance of sea level due to underwater earthquakes using the deformation model once the seismic fault parameters such as earthquake location (longitude, latitude, depth), magnitude, fault geometry (strike, dip, rake, length and width) and slip, are obtained. The Okada deformation model [21] is used in the current study. Propagation is the flow of this displaced volume of sea surface water under gravity towards the coast. An SWE solver simulates propagation [27] and demonstrates the ADCIRCv55 capability for global simulation of storm tides for two intense storms, Hurricane Katrina and Super Typhoon Haiyan. This article compared ADCIRCv55 simulations with real-world data for astronomical and storm tides induced by hurricanes; the results demonstrated good agreement, validating the model’s accuracy and reliability. Both tsunami and storm surges are shallow-water gravity waves, although their generation and forcing conditions differ [24]. The current article makes use of ADCIRCv55 to simulate the propagation of tsunamis. This current version of ADCIRC can be downloaded from https://github.com/adcirc. ADCIRC solves the SWEs numerically by discretizing the continuous equations on an unstructured mesh with the Finite E method [25]. ADCIRCv55 is the apt choice for the current work compared to its previous versions. V55 utilizes an accurate modified form of the SWE in spherical coordinates, capturing the essential tide and wave dynamics in deep and shallow waters with varying scales in larger domains like the entire globe [27]. To accurately compute the governing equations on the entire globe, the model formulation and the code were enhanced [27]. This improvement includes rotating the Earth to eliminate pole singularities. V55 introduces new numerical techniques that remove gravity wave-based (Courant–Friedrichs–Lewy) stability constraints, which allows for larger computational time steps, significantly boosting simulation speed without compromising accuracy. The newly introduced semi-implicit time integration scheme is computationally effective and is 1–2 times faster than the previous versions of ADCIRC.

The quality and design of the unstructured mesh play a crucial role in successfully solving the SWEs with ADCIRC. The global unstructured mesh in the current study is generated using an open-source toolbox OceanMesh2D 3.0.0 (Robert *et al.*, 2019). OceanMesh2D is a specialized software based on MATLAB that generates ocean meshes compatible with various ocean models, including ADCIRC. The software has a user-friendly interface and comprehensive documentation with a script-driven automatic mesh generation approach. This automated approach ensures consistency and efficiency without manual editing, saving time and reducing potential errors while creating complex meshes. It integrates with various data sources, importing bathymetry and coastline information to guide the mesh generation process. It also accurately captures complex coastlines and bathymetry, leading to more realistic wave propagation and inundation simulations. OceanMesh2D supports adaptive mesh resolution by meshing with smaller triangles (fine resolution) in the coastal waters and employing larger triangles (coarse resolution) in deeper waters; this adaptive resolution optimizes computational resources, leading to faster simulations. The global mesh for global tsunami simulations in the current article is built in a stereographic projection centred at the North Pole. This projection preserves angles on the sphere, making it well-suited for accurately representing a realistic global ocean. The mesh seamlessly wraps around the entire Earth, including an element placed over the North Pole. This avoids artificial discontinuities at the poles, which can cause simulation issues. The unstructured triangular mesh on the spherical earth accurately conforms to the coastline and covers a spatial resolution range of 2–20 km. The OceanMesh2D inbuilt full-resolution Global Self-consistent Hierarchical High-resolution Shorelines (GSHHS) dataset [28] is used to define the shoreline boundary while generating the mesh. This toolbox can be downloaded from https://github.com/CHLNDDEV/OceanMesh2D.

**Figure 1.**
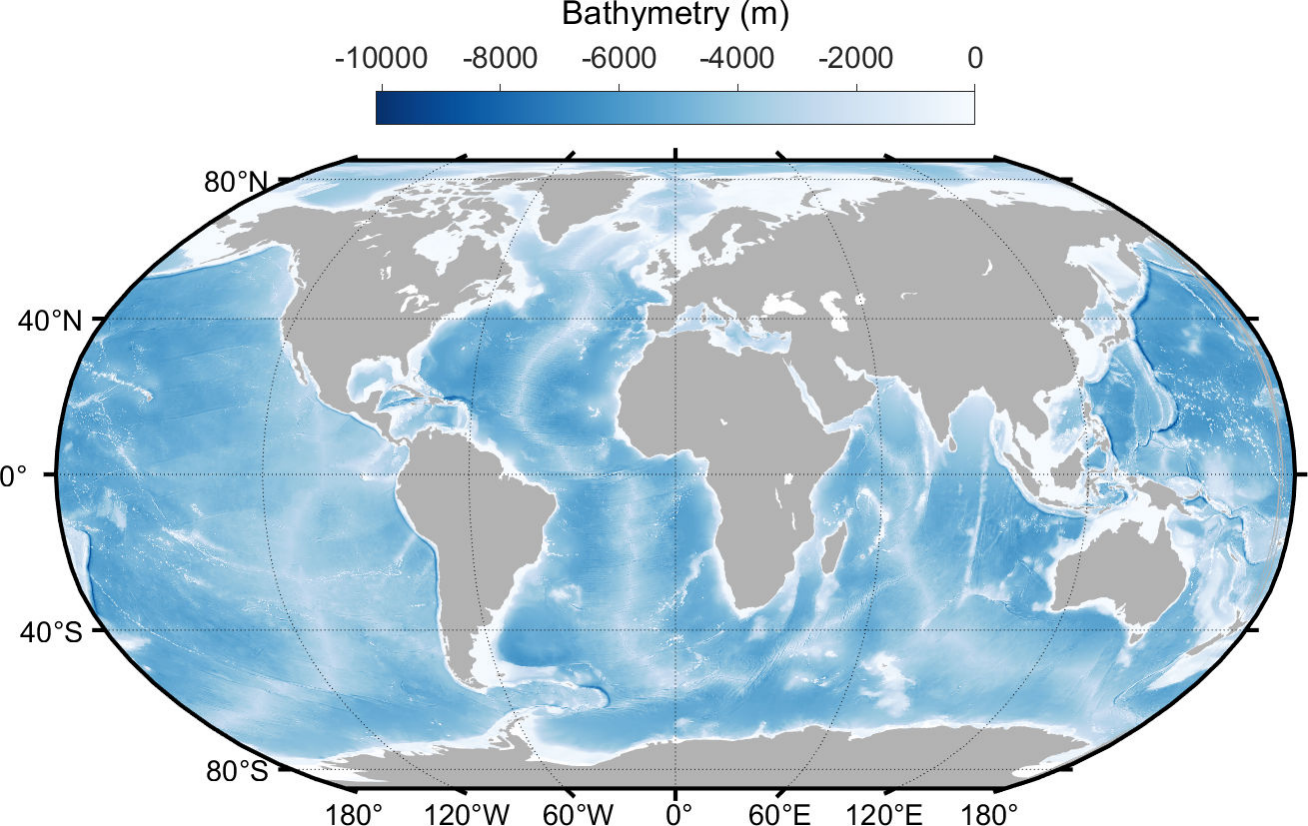
Global model domain (on Robinson projection) and bathymetry.

**Figure 2.**
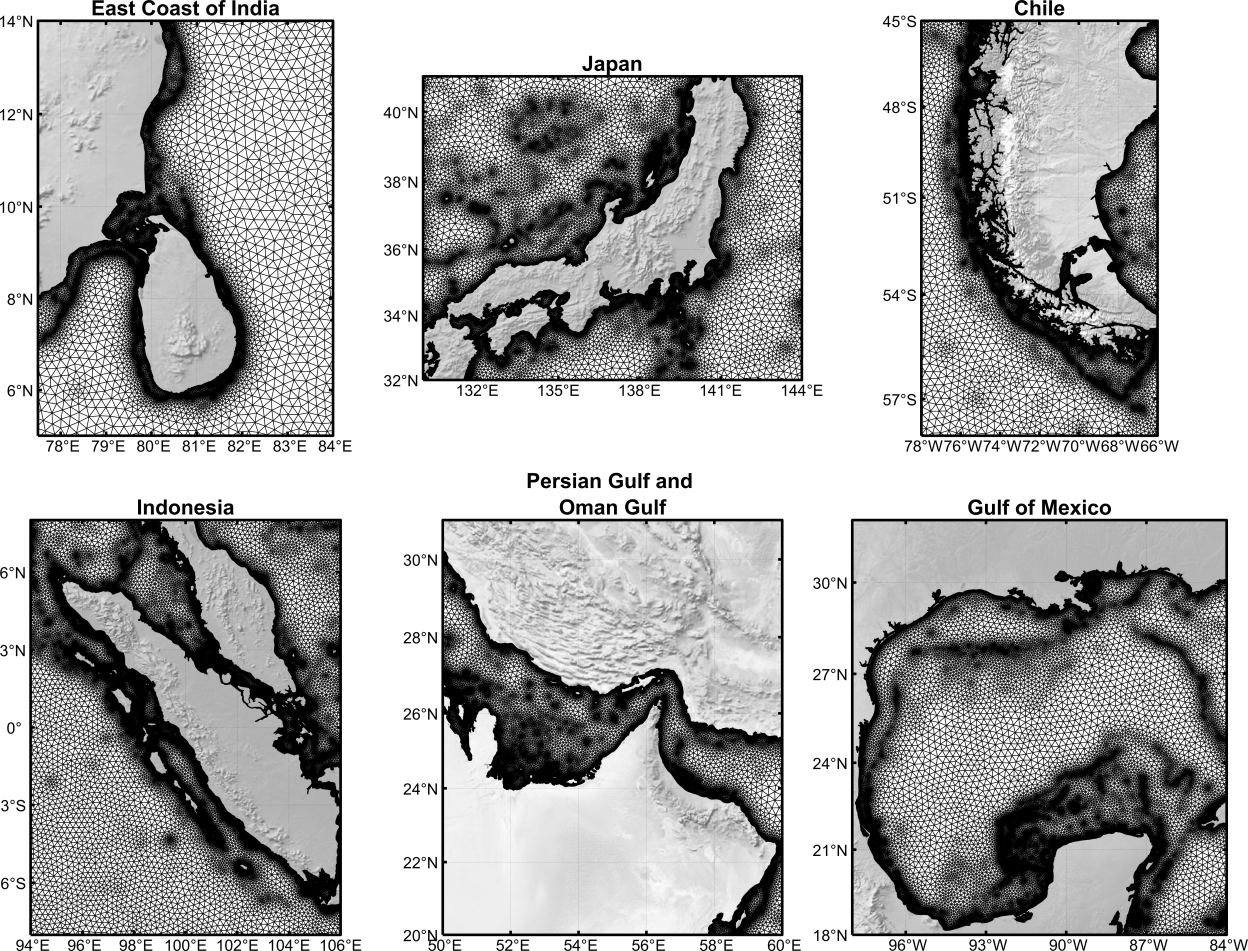
Triangular gridded mesh (on Mercator projection) for various parts of the global mesh.

The accuracy and quality of bathymetric data are critical for generating unstructured meshes using OCEANMESH2D for ADCIRC simulations. The present study’s bathymetric data is sourced from the General Bathymetric Chart of the Oceans (GEBCO) [29] dataset. The GEBCO comprises an international group of experts in ocean mapping who aim to provide the most authoritative publicly available bathymetry of the world’s oceans. GEBCO provides a high-resolution global grid with 30 arc-second spacing, widely regarded as one of the most comprehensive and accurate global bathymetric datasets available. This 30 arc-sec data from GEBCO is used for the bathymetry for the global domain. Figure 1 illustrates the global model domain along with bathymetry on a Robinson projection generated using OceanMesh2D and the bathymetry employed in the current study. The mesh consists of 3 458 668 nodes and 6 628 428 triangular elements. The global mesh resolution varies depending on the depth of the water. The mesh has a fine resolution of 2 km in shallow areas near coastline waters, where capturing accurate wave behaviour is crucial. In deeper waters further away from land, the mesh coarsens to 20 km. This optimization balances accuracy with computational efficiency, ensuring the model can simulate vast stretches of the ocean without exceeding pre- and post-processing limitations. Figure 2 displays an unstructured mesh (on Mercator projection) over a few complex coastal regions of different oceans, depicting the fineness of the mesh.

In the current study, we have utilized a global unstructured mesh with variable spatial resolution, with a finer resolution of 2 km in shallow waters and a coarser resolution of up to 20 km in deep ocean waters. This variable resolution is critical for capturing the different characteristics of tsunami waves in deep water versus shallow water. The rationale for this variable resolution is to capture detailed nearshore processes, where finer resolution is essential for accurately modelling wave propagation while maintaining computational efficiency in the deep ocean, where local bathymetric features has less influence on tsunami dynamics. Tsunami waves have very long wavelengths (often exceeding 100 km) in deep waters, where the ocean’s depth primarily influences the wave speed. In such regions, a coarser resolution (20 km) is sufficient because the wave characteristics do not require fine-scale bathymetric detail to be accurately resolved. The long wavelength means large grid cells can capture the wave dynamics without compromising accuracy while ensuring computational efficiency. As tsunami waves approach the coastline, their wavelength shortens significantly due to the shallower depth, leading to a complex wave behaviour such as amplification. In these coastal regions, finer mesh resolution (2 km) is essential to accurately resolve the shorter wavelengths and the interaction of the wave with complex coastal topography. A finer resolution ensures that the steep gradients in bathymetry near the shore are well-represented, allowing the model to accurately capture the amplification and run-up processes. The resolution choices in the current work align with those discussed in [27], where a similar resolution range (~2−20 km) was used in global ocean models for storm tide simulations.

**Figure 3.**
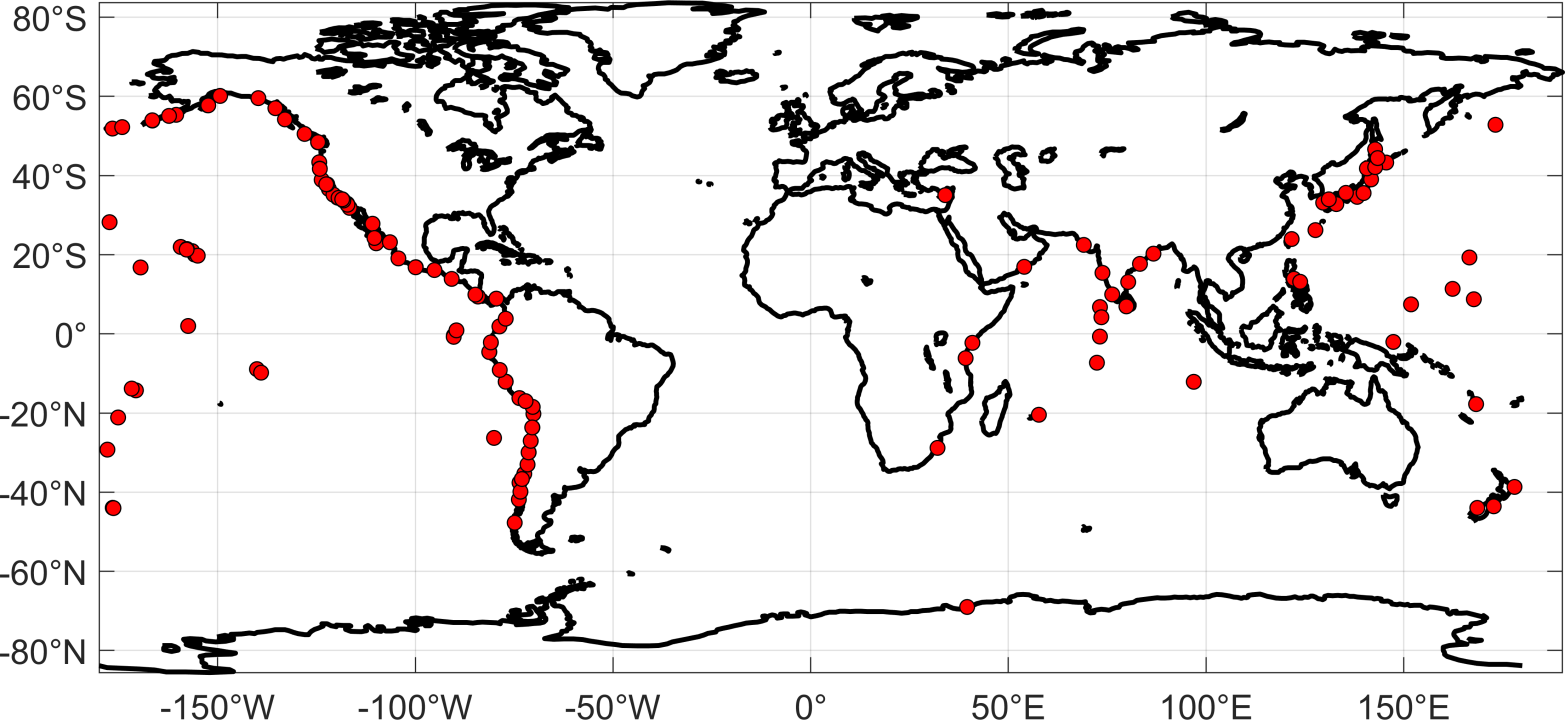
Observation locations (obtained from IOC sea level monitoring facility) used to validate the model simulations. (The details of the observations shown in table 1.) An interactive figure of the same can be viewed online at the link: https://public.tableau.com/views/TidegaugeobservationsfromtheIOC-sealevel/Dashboard1?:language=enUS&publish=yes&:sid=&:display_count=n&:origin=viz_share_link.

The ADCIRCv55 model was run on 480 Intel Sandy Bridge Processors on the HPC system, and its run time was about 10 min for 30 h of model simulation for propagation using 1296 processors. This article used a time step of 2 s and a quadratic bottom friction of value 0.028 to simulate tsunami propagation. The ADCIRC model solves the generalized wave continuity equation, which introduces a numerical parameter, TAU0, that weights the SWE. In the current simulations, a value of TAU0 = 0.05, constant in space and time in line with [30] is taken to conserve the mass (electronic supplementary material, figure S3). The model outputs wave heights at every grid point at a specified time interval. The simulated wave heights for the entire grid are saved for every five min, and these wave heights are compared against available tide gauge observations. The computation time can be further optimized to five min if the output is requested only at the coastal focal points instead of all the grid points. The tide gauge observations are extracted from the website https://www.ioc-sealevelmonitoring.org/ [31]. The Sea Level Station Monitoring Facility (SLSMF) is a website developed and maintained by the Flanders Marine Institute (VLIZ) for the Intergovernmental Oceanographic Commission (IOC) [32] of the United Nations Educational, Scientific and Cultural Organization (UNESCO). The SLSMF provides worldwide information about the data and the operational status of real-time sea level monitoring stations. It is an important tool for monitoring sea level changes, providing tsunami warnings and protecting coastal communities. The SLSMF is a valuable resource for scientists, policymakers and the public. The locations of various observations used in the current study are shown in figure 3 (on Mercator projection), and the respective locations, along with their code and country names, are given in table 1. The tide gauge observations from the IOC sea level were collected from various locations where wave height was significant (≥0.5 m) due to tsunami, and residuals were computed. Residuals were computed by removing astronomical tide components from the tide gauge records to obtain the water levels solely due to the tsunami wave. These residuals were computed using UTIDE [33] and compared with the model computed water levels due to tsunami at available tide gauge locations. Computed travel times were also validated using available observations. The use of UTIDE, a robust tidal analysis and prediction tool, was instrumental in isolating the tsunami signal from the background noise of tidal fluctuations within the IOC sea level data. This detiding process ensured a more accurate comparison with model simulations, which inherently exclude tidal influences.

**Table 1.** Tide gauge observations from the IOC sea level link used in the various tsunami events (https://www.ioc-sealevelmonitoring.org/list.php). The locations of the observations are shown in figure 3.

code	lon (deg)	lat (deg)	country	location
acap	−99.917	16.833	Mexico	Acapulco
acya	−99.903	16.838	Mexico	Acapulco Club de Yates
adak	−176.632	51.863	USA	Adak
anto	−70.405	−23.654	Chile	Antofagasta
aren	−123.705	38.913	USA	Arena Cove
aric	−70.323	−18.476	Chile	Arica
atic	−73.694	−16.231	Perú	Atico, Arequipa
balt	−90.283	−0.433	Ecuador	Baltra,Galapagos
cald	−70.825	−27.065	Chile	Caldera
call	−77.167	−12.069	Perú	Callao, La-Punta
char	−124.322	43.345	USA	Charleston, OR
chit	−176.369	−44.025	New Zealand	Owenga, Chatham Island
const	−72.458	−35.356	Chile	Constitucion
coqu	−71.335	−29.95	Chile	Coquimbo
corr	−73.427	−39.887	Chile	Corral
cres	−124.183	41.745	USA	Crescent City
dutc	−166.533	53.883	USA	Dutch Hbr Unalaska
hana	145.57	43.28	Japan	Hanasaki
hens	−133	54.183	Canada	Henslung Cove
hiva	−139.034	−9.805	France	Hiva Oa (Marquesas, French Polynesia)
hono	−157.867	21.307	USA	Honolulu,Oahu
iqui	−70.148	−20.205	Chile	Iquique
kors	142.767	46.633	Russia	Korsakov
kwaj	167.738	8.737	Marshall Islands	Kwajalein
lali	−80.906	−2.218	Ecuador	La Libertad
lebu	−73.664	−37.594	Chile	Lebu
lomb	147.374	−2.042	Papua New Guinea	Lombrum Manus Is
manz	−104.298	19.064	Mexico	Manzanillo
midx	−177.356	28.207	USA	Midway
naha	127.67	26.21	Japan	Naha
nawi	−159.36	21.957	USA	Nawiliwili, Kauai
nkfa2	−175.197	−21.13	Tonga Island	Nuku'Alofa Harbour wharf
nuku	−140.085	−8.915	France	Nuku Hiva (Marquesas, French Polynesia)
omae	138.22	34.61	Japan	Omaezaki
pago	−170.691	−14.277	Samoa	Pago Pago
pagx	−170.691	−14.277	Samoa	Pago Pago
quepo	−84.17	9.425	Costa Rica	Quepos
sanf	−80.109	−26.292	Chile	San Felix
sant	−90.307	−0.752	Ecuador	SantaCruz, Galapagos
sdpt	−160.502	55.337	USA	Sand Point
sumt	172.773	−43.57	New Zealand	Christchurch
talc	−73.106	−36.701	Chile	Talcahuano
tosa	132.96	32.78	Japan	Tosashimizu
valp	−71.628	−33.028	Chile	Valparaiso
vanu	168.308	−17.755	Vanuatu	Port Vila
wait	−176.561	−43.946	New Zealand	Waitangi, Chatham
wint	−128.03	50.51	Canada	Winter Harbour
xmas	−157.473	1.984	Kiribati	Christmas
ancu	−73.833	−41.867	Chile	Ancud
atka	−174.173	52.232	USA	Atka, AK
cabo	−109.91	22.879	Mexico	Cabo San Lucas
fpnt	−122.465	37.807	USA	Ft Point, San Fran
gist	178.023	−38.675	New Zealand	Gisborne
kahu	−156.472	20.898	USA	Kahului, Maui
kawa	−155.832	20.036	USA	Kawaihae, Hawaii
kgak	−162.324	55.059	USA	King Cove
kodi	−152.512	57.732	USA	Kodiak Island, AK
lajo	−117.258	32.867	USA	La Jolla
losa	−118.272	33.719	USA	Los Angeles
mont	−121.887	36.605	USA	Monterey
ofun	141.75	39.02	Japan	Ofunato
rfrt	−177.904	−29.251	New Zealand	Raoul Island Fishing Rock
sanb	−119.685	34.408	USA	Santa Barbara
sand	−117.173	32.713	USA	San Diego
sanm	−118.5	34.008	USA	Santa Monica
sewa	−149.427	60.119	USA	Seward
sped	−74.883	−47.717	Chile	Isla San Pedro
upol	−171.761	−13.827	Samoa	Apia Upolu
wake	166.618	19.29	USA	Wake
blueb	57.711	−20.444	Mauritius	Blue Bay
chenn	80.3	13.1	India	Chennai
cocb	96.892	−12.117	Australia	Cocos Island
coch	76.26	9.96	India	Cochin
colo	79.85	6.941	Sri Lanka	Colombo
ganm	73.152	−0.687	Maldive Islands	Gan
garc	72.394	−7.29	UK	Diego Garcia
hani	73.167	6.767	Maldive Islands	Hanimadhoo
jack	168.616	−43.973	New Zealand	Jackson Bay
lamu	40.9	−2.267	Kenya	Lamu
male	73.527	4.19	Maldive Islands	Male
marm	73.8	15.41	India	Marmagao
para	34.037	35.038	Cyprus	Paralimni
sala	54.007	16.935	Oman	Salalah
syow	39.57	−69.008	Antarctica	Syowa
vish	83.28	17.68	India	Visakhapatnam
zanz	39.183	−6.15	Tanzania	Zanzibar
okha	69.08	22.47	India	Okha
rich	32.078	−28.811	South Africa	Richards Bay
yaku	−139.733	59.55	USA	akutat
sitk	−135.333	57.05	USA	Sitka
neah	−124.617	48.3667	USA	Neah Bay
pslu	−120.733	35.1667	USA	Port San Luis
rinc	−119.433	34.35	Rincon Island	Rincon Island
ense	−116.633	31.85	Mexico	Ensenada
tala	−81.2833	−4.5833	Perú	Talara
mata	−72.1167	−17	Perú	Matarani,Arequipa
moku	−157.8	21.4333	USA	Mokuoloe, Oahu
alam	−122.298	37.772	USA	Alameda
chuu	151.85	7.45	Micronesia	Chuuk
chimb	−78.6611	−9.1278	Perú	Chimbote, Ancash
San Cristobal	−89.62	0.8833	San Cristobal	San Cristobal
tumc	−78.75	1.8722	Colombia	Tumaco
buve	−77.15	3.8278	Colombia	Buenaventura
Naos Island	−79.5278	8.8944	Naos Island	Naos Island
Puntarenas	−84.82	9.9167	Puntarenas	Puntarenas
prsj	−90.82	13.883	Guatemala	Puerto San José
sali	−95.18	16.117	Mexico	Salina Cruz, Oax.
maza	−106.45	23.15	Mexico	Mazatlán
Guaymas	−110.85	27.817	Guaymas	Guaymas
lpaz2	−110.35	24.217	Mexico	La Paz2
Womens Bay	−152.406	57.7278	Womens Bay	Womens Bay
Massacre Bay	173.2167	52.8167	Massacre Bay	Massacre Bay
hilo	−155.061	19.75	USA	Hilo, Hawaii
john	−169.517	16.7722	USA	Johnston
Eniwetok	162.3722	11.3611	Eniwetok	Eniwetok
Hondagua	122.2167	13.9278	Hondagua	Hondagua
lega	123.8056	13.15	Philippines	Legaspi
thua	121.65	23.95	Taiwan	Hualien
Sasebo	129.6278	33.1389	Sasebo	Sasebo
Moji	131.05	33.9833	Moji	Moji
Maizuru	135.38	35.617	Maizuru	Maizuru
Tsukizi	139.85	35.5722	Tsukizi	Tsukizi
hako	140.6833	41.7944	Japan	Hakodate
Urakawa	142.7833	42.1167	Urakawa	Urakawa
Monbetsu	143.4056	44.4056	Monbetsu	Monbetsu

*Note*: An interactive version of the above table can be viewed in the link: https://public.tableau.com/views/TidegaugeobservationsfromtheIOC-sealevel/Dashboard1?:language=en-US&publish=yes&:sid=&:display_count=n&:origin=viz_share_link

## 4. Performance evaluation

For tsunami warning centres, accurate prediction of both the first peak arrival time and its amplitude of a tsunami are critical for issuing warnings towards saving lives and minimizing damage [34]. Timely warnings issued based on the predicted arrival time and first peak amplitude allow the coastal communities to evacuate to safe zones. In essence, accurate predictions of these two factors are fundamental components of tsunami warning systems, strengthening coastal community preparedness and response strategies. First, these factors are calculated from the observations and the model and represented in a plot, and the model’s performance on these aspects is discussed. Second, several statistical metrics are employed to quantify the accuracy and reliability of the ADCIRC model simulations against the observations for the entire simulation length. Regression (R) analysis is conducted to explore the relationship between the observed heights and model wave heights. The statistical significance (SS) in percentage is also shown along with R. The normalized root mean square deviation (NRMSD) is used to assess the overall deviation of the simulated values from the observed values, providing a dimensionless measure that facilitates comparison across different scales of values. The normalized mean absolute deviation (NMAD) quantifies the average normalized absolute difference between predicted and observed values, which is crucial for understanding worst-case scenarios. The normalized Nash-Sutcliffe efficiency (NNSE) is also calculated to evaluate the model’s performance relative to the mean observed data. The metrics used for model performance evaluation are detailed in table 2, along with the minimum, maximum and best possible values for each performance metric, along with the respective formulae. Though we have used the above statistical metrics, it should be noted that there will be mismatches between the simulated model results and observations, particularly in terms of arrival times. This discrepancy can negatively affect metrics like R, which may appear less favourable due to timing mismatches, even when the overall model performance is good.

**Table 2.** Statistical performance metrics.

performance metric	abbreviation	range	best value	formula
regression	R	[−1, 1]	1	$\frac{\sum \left(O_{i}-\overset{-}{O}\right)\left(p_{i}-\overset{-}{p}\right)}{\sqrt{\sum {\left(O_{i}-\overset{-}{O}\right)}^{2}\sum {\left(p_{i}-\overset{-}{p}\right)}^{2}}}$
root mean square deviation	RMSD	[0, ∞)	0	${\sqrt{\frac{1}{n}\sum {\left(o_{i}-p_{i}\right)}^{2}}}^{,}$
normalized root mean square deviation	NRMSD	[0, ∞)	0	$\frac{RMSD}{\max\left(o\right)-min\left(o\right)}$
mean absolute deviation	MAD	[0, ∞)	0	$\frac{1}{n}\sum \left|o_{i}-p_{i}\right|$
normalized mean absolute deviation	NMAD	[0, ∞)	0	$\frac{MAD}{\max\left(o\right)-min\left(o\right)}$
Nash-Sutcliffe efficiency	NSE	(∞, 1]	1	$1-\frac{\sum {\left(o_{i}-p_{i}\right)}^{2}}{\sum {\left(o_{i}-\overset{-}{o}\right)}^{2}}$
normalized Nash-Sutcliffe efficiency	NNSE	(0, 1]	1	$\frac{1}{2-NSE}$

*Note*: *o*_i_ and *p*_i_ denote the observed and simulated wave heights respectively; and are the mean of observed and simulated wave heights; max and min are the maximum and minimum of the value.

Discrepancies between ADCIRC model predictions and tide gauge data, particularly in wave heights and arrival times, arise from factors like bathymetric data limitations, fault parameters, numerical model assumptions and aftershocks. These factors are discussed below in brief.

1. *Bathymetry*: The resolution and accuracy of the bathymetry, particularly in coastal and shallow water regions, play a crucial role in wave propagation and amplification. The bathymetric data used (from the GEBCO dataset) is of high resolution but may contain inaccuracies in certain nearshore regions due to limited surveys. These inaccuracies, if any, can result in local errors in wave height prediction, either amplifying or reducing the model’s estimates.
2. *Initial seismic fault parameters*: The accuracy of tsunami generation relies heavily on the initial fault parameters, such as slip, rupture length and depth. Minor uncertainties in these parameters, which are derived from seismic data, can affect the initial sea surface displacement and, consequently, the tsunami wave characteristics. For example, variations in the estimated slip distribution across the fault can lead to over- or underestimation of the wave heights, especially near the source.
3. *Numerical model*: The ADCIRC model solves the SWE, which, while effective for long-wave propagation, does not account for dispersive effects or water compressibility. In deep waters, these effects are negligible, but as tsunamis approach the coast, neglecting dispersive behaviour can lead to early wave arrival times. Studies like [35] have shown that frequency dispersion and water compressibility can slightly delay wave propagation, explaining why arrival times are sometimes predicted earlier than observed.
4. *Exclusion of aftershocks and secondary waves*: The current set-up does not incorporate aftershocks, which can generate additional waves following the main event. In several historical tsunamis, aftershocks contributed significantly to secondary wave heights, particularly in the near-field region. This omission could explain some underestimations of wave heights in certain locations, particularly after the initial wave.
5. *Local coastal morphological features*: Coastal morphology, such as the presence of bays, inlets and steep topography, can significantly influence wave amplification. While ADCIRC’s mesh resolves coastal features, there may still be some unresolved finer-scale morphological effects due to mesh resolution constraints that lead to discrepancies between predicted and observed wave heights. In regions with complex coastal geometries, these effects could cause the model to either overestimate or underestimate wave heights depending on local amplification factors.

The observed discrepancies are likely due to a combination of uncertainties in seismic source data, limitations of the bathymetric resolution, simplifications in the SWE and the exclusion of aftershocks and certain local morphological effects.

## 5. Results and discussion

In the current section, five major global tsunamis are simulated using the methodology discussed, and the results are compared with the tide gauge observations. The tsunamigenic seismic events considered are shown in table 3. To issue tsunami warnings against a generated earthquake, the maximum wave height and the arrival time of this peak wave height are paramount for issuing adequate warnings and implementing evacuation measures. The maximum elevation and travel time plots are shown for each simulation, considering their importance in real-time warnings. The statistical performance metrics obtained for each simulation are shown in electronic supplementary materials, tables.

**Table 3.** Fault parameters used for computing the initial deformation.

event	date & time (UTC)	magnitude (Mw)	long (^o^E)	lat (^o^N)	depth (km)	strike (^o^)	dip (^o^)	rake (^o^)	length (km)	width (km)	slip (m)	reference
Tohoku-Oki, Japan	11-Mar−2011 05:46:00	9.00	142.34	38.30	28.04	202.0	12.0	78.5	600.0	210.0	41	[36]
Maule, Chile	27-Feb−2010 06:34:23	8.90	−72.72	−35.85	37.0	17.5	18.0	113.18	600	187	13	[37]
Sumatra, Indonesia	26-Dec−2004 00:58:50	9.25	94.57	3.83	25.0	323.0	12.0	90.00	220	130	18	[2]
			93.90	5.22	25.0	348.0	12.0	90.00	150	130	23	
			93.21	7.41	25.0	338.0	12.0	90.00	390	120	12	
			92.60	9.70	25.0	356.0	12.0	90.00	150	95	12	
			92.87	11.70	25.0	10.0	12.0	90.00	350	95	12	
Alaska, North America	28-Mar−1964 03:36:00	9.20	−147.73	61.00	25.0	237.0	12.0	45.00	50	50	17	Ichinose *et al.*, 2007
Valdivia, Chile	22-May−1960 19:11:20	9.50	−73.40	−38.14	25.5	7.0	20.0	110.00	1000	250	40	[38]

### 5.1. Japan tsunami event 11 March 2011

**Figure 4.**
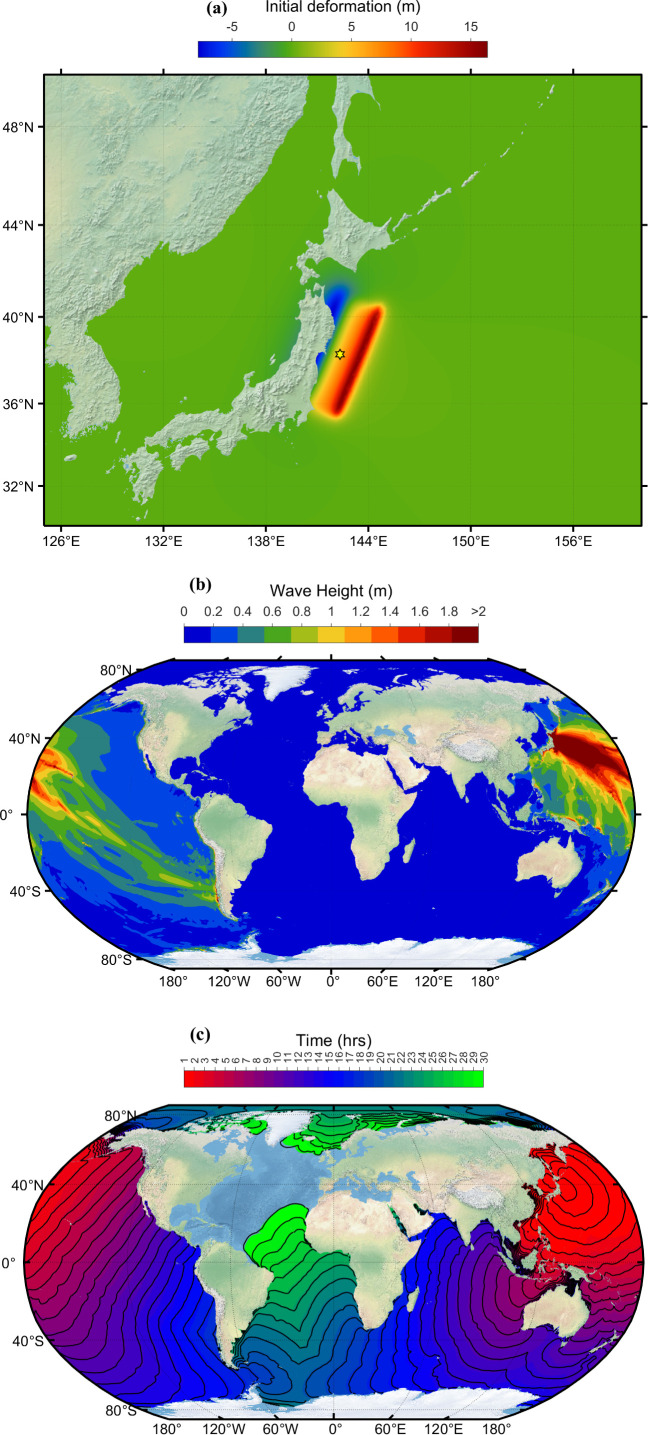
(*a*) Initial deformation of sea level. (*b*) Simulated maximum wave amplitude distribution. (*c*) Travel time contours of tsunami wave due to Japan tsunami event 11 March 2011. (The figure is on Robinson projection.)

**Figure 5.**
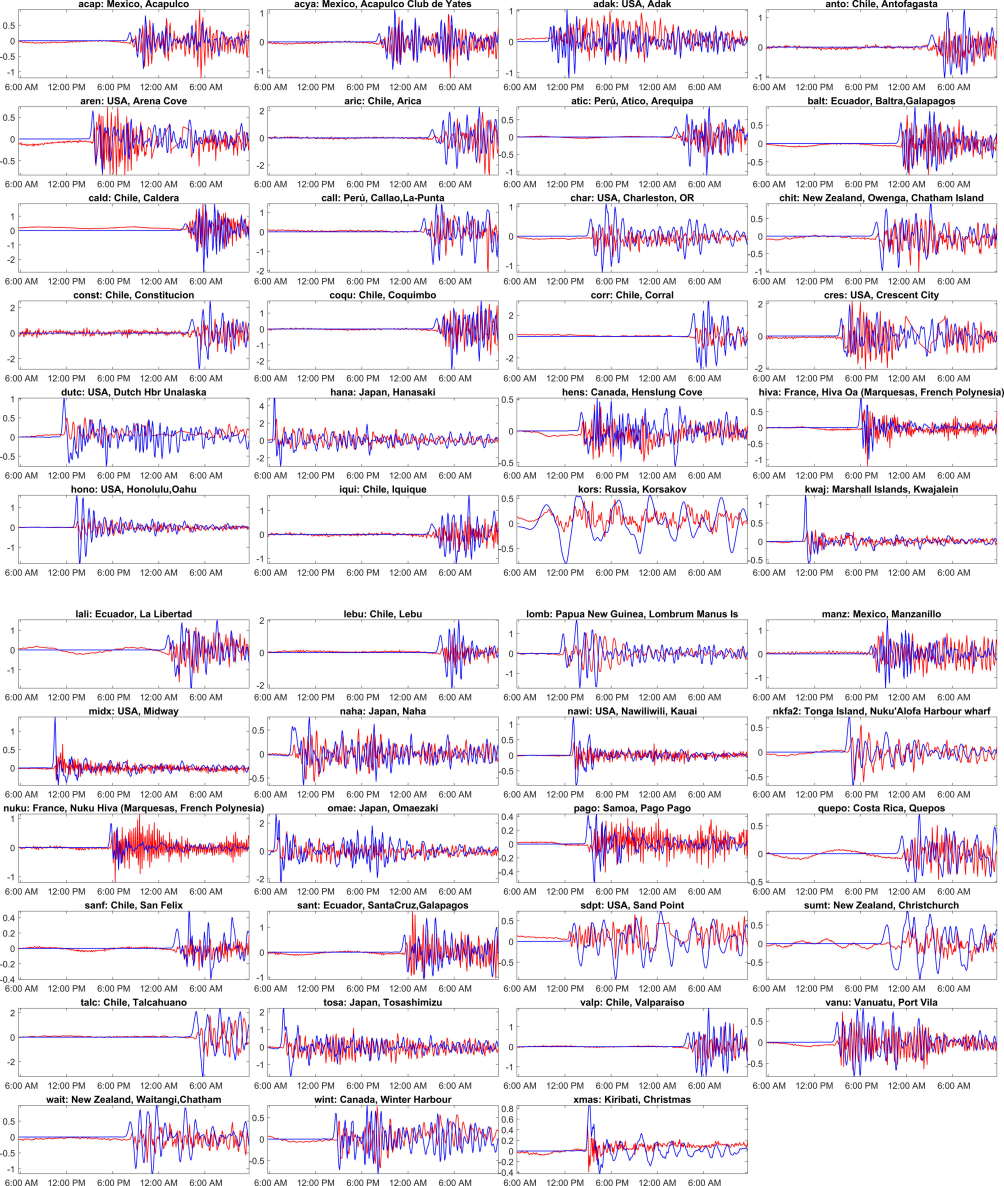
Comparison of the computed tsunami wave heights against the tide gauge observations at various global locations for the Japan 2011 event. The red colour corresponds to tide gauge observations, and the blue colour represents a numerical simulation. The locations of the tide gauge stations shown as titles on each subplot are marked in figure 3 and shown in table 1. The *x*-axis represents the time stamp, and the *y*-axis represents the wave height amplitude in meters. The starting time in the abscissa in each subplot is 11-Mar−2011 05:46:00 UTC. (See electronic supplementary material, table S3, for statistical performance metrics.)

On 11 March 2011, at 05:46 UTC (local time 14:46), a subduction megathrust earthquake with a moment magnitude of 9 Mw occurred 30 km below the ocean floor off the northeastern coast of Honshu, Tohoku, Japan [36]. This is Japan’s strongest earthquake ever recorded and the fourth strongest globally since 1900. This earthquake is also called the 2011 Tohoku Earthquake or the Great East Japan Earthquake. The earthquake caused widespread destruction on the land and triggered a giant tsunami that devasted many coastal regions of Japan [39]. This massive earthquake and subsequent tsunami caused severe damage to the Fukushima Daiichi nuclear power plant, leading to meltdowns, hydrogen–air explosions and the release of significant radiation leaks. Hundreds of thousands of people were evacuated due to the nuclear threat. It was the most severe nuclear disaster since the Chernobyl disaster in 1986, which challenged the nuclear communities across the world to reassess nuclear power safety measures [40]. Figure 4*a* shows the computed initial deformation for the 2011 Japan earthquake. The focal fault parameters used for generating this initial deformation are taken from [36] and are shown in table 3. The maximum simulated tsunami wave height distribution over the entire simulation is displayed in figure 4*b*. Travel time contours of tsunami waves at every 1 h interval are shown in figure 4*c*. Figure 5 compares observations and model-simulated water levels at different locations over the globe. All these observations are taken from the IOC sea level website and are plotted in alphabetic order based on the station ID. The observations are averaged to five min from one min and displayed in figure 5. The tide gauge ID, country and location name are given as titles on each subplot, which can be accessed from table 1 for further information. From figure 5, the blue curves representing the ADCIRC simulations generally track the red curves representing the observations fairly close. This suggests that the model is doing a good job of capturing the timing and general shape of the tsunami waves for most of the stations. It can be observed from figure 5 that at most of the stations, the peak tsunami wave is slightly overestimated, and the wave arrival is a few minutes (about 10 min) earlier. One can compromise with this kind of model behaviour from an operational perspective as this leads to early evacuation measures, and timings can be taken sooner. There are some exceptions where the blue curves diverge from the red curves. For example, at the core, dutc, cebu, lomb, sand, etc. stations, the model predicts a larger wave than the observed one. This suggests that the model may not be predicting the exact observed wave height, but it is still doing a good job of approximating reality in most cases. A possible reason for the mismatch could be because of the uncertainties involved in the generation mechanism of initial deformation [41]. Discrepancies in bathymetry [42,43] and/or the bottom friction used at the location can also result in these mismatches, as the accuracy of the wave computations largely depends on the accuracy of these parameters. Similarly, the underestimation after the initial peaks can be noticed in some instances. A possible reason for this might be that the simulations do not include aftershocks from the actual earthquake. Japan experienced over 1000 aftershocks since the earthquake, with 80 registering over magnitude 6.0 Mw and several of which have been over magnitude 7.0 Mw. A magnitude 7.4 Mw at 15:08 (JST), 7.9 Mw at 15:15 and a 7.7 Mw quake at 15:26 all occurred on 11 March. While modelling tsunamis, incorporating these aftershocks can lead to an accurate estimation of wave heights over the entire simulation period. As aforementioned, discrepancies in the bathymetry data may also contribute to the mismatch in these instances, too.

The Japan Meteorological Agency (JMA) has issued the most severe tsunami warning, rating it as a major tsunami. A tsunami hit Japan’s coast within an hour (figure 4*c*) of the earthquake. From figure 4*b*, it can be seen that the northeastern part of Japan’s coast (refer figure 5 at hana, omae locations) faced tsunami wave heights greater than 2 m, and southeastern part (refer figure 5, tosa location) had tsunami wave heights greater than 1 m. Figure 4*b* depicts that the tsunami wave heights on the west coast of Japan were less than 1 m. A similar result was published by JMA [44]. The city of Sendai, the capital of Miyagi Prefecture, was closest to the epicenter (130 km away), was hard hit and experienced a severe tsunami wave height greater than 7.6 m [44]. The current model predicted it to be 9.3 m. At the Fukushima Daiichi nuclear power plant, JMA published wave height to be greater than 9.3 m [44]; the current model predicted it to be 14.4 m. Figure 4*b* shows that the tsunami propagated throughout the Pacific Ocean region, reaching the entire Pacific coast of North and South America from Alaska to Chile. The wave reached the North American coasts in about 10–12 h and the South American coast in about 17–20 h, which can be visualized in figure 4*c*. Tsunami warnings were issued, and evacuations were carried out in many countries bordering the Pacific. The tsunami heights resulting from the earthquake were measured across various locations globally. In New Zealand, the Chatham Islands (figure 5; chit, wait) and New Zealand’s coast (figure 5; sumt) experienced a 0.5 m tsunami. In North America, Canada (figure 5; hens, wint) recorded heights greater than 0.5 m, the USA (figure 5; aren, char, cres) saw tsunami height exceeding 1 m, Mexico (figure 5; acap, acya, manz) reported a 1 m tsunami. Costa Rica (figure 5; qepo) observed a 0.5 m tsunami. In South America, Ecuador (figure 5; lali) faced a tsunami exceeding 1 m, while Peru (figure 5; atic, call) recorded heights surpassing 0.5 m. Chile experienced varying tsunami heights in different locations, with Antofagasta (figure 5; anto) at 1 m, Arica (figure 5; aric) at 2 m and several other locations (figure 5; cald, const, coqu, corr, iqui, lebu, talc, valp) reporting heights ranging from 1 to 2 m. Russia’s island of Kors (figure 5; kors) witnessed a 0.5 m tsunami. Among the USA islands, Adak (figure 5; adak) experienced a 1 m tsunami, and several others (figure 5; dutc, hono, nawi, midx, sdpt) reported tsunamis of greater than or equal to 0.5 m. Additionally, the Chilean island of Sanf (figure 5; sanf) faced a 0.5 m tsunami, while Ecuador’s islands, including Balt (figure 5; balt) and Sant (figure 5; sant), reported heights exceeding 0.5 and 1 m, respectively.

**Figure 6.**
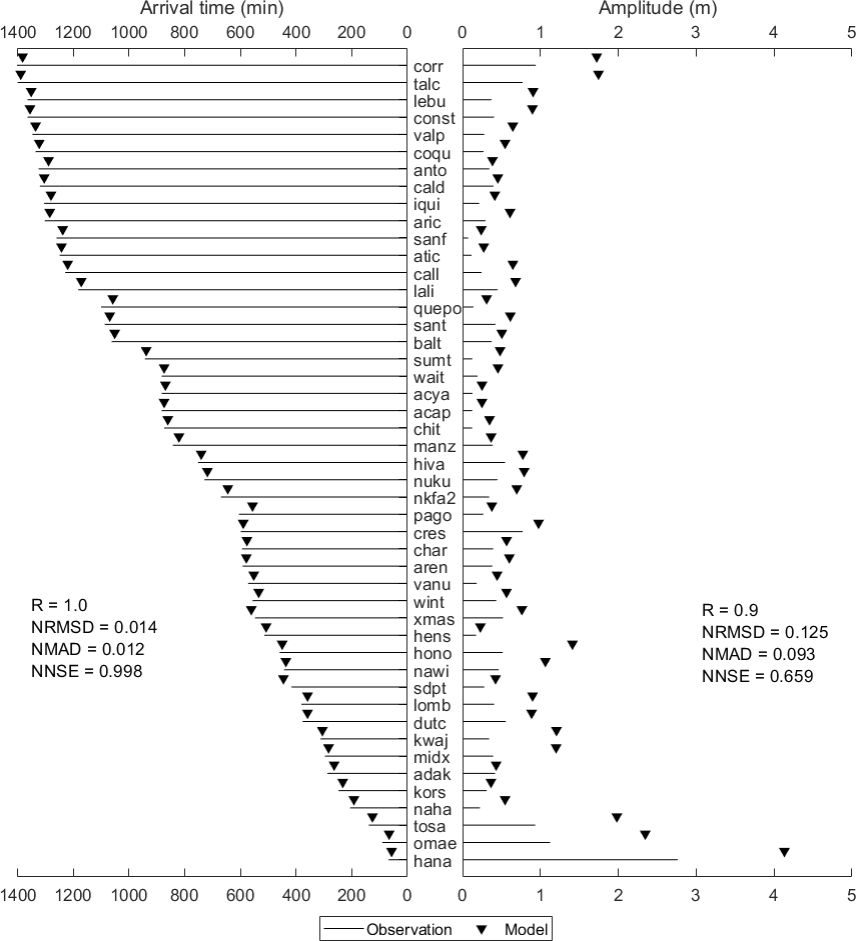
Comparison of observed and simulated first peak values for travel time and amplitude for the Japan tsunami event of 11 March 2011. The location of each station is indicated in figure 3, and the details are shown in table 1.

Figure 6 compares observed and simulated values for the amplitude of the first peak and its arrival time at various stations shown in figure 5 for the 2011 Japan tsunami event. The stations in figure 6 (bottom to top) are sorted by distance from the epicentre. The model predicted much larger amplitudes for nearby stations (hana, omae and losa), possibly because of the used tsunami’s initial deformation. From figure 6, it is evident that the differences in amplitude range from −1.37 m at hana to 0.02 m at manz, with most values being negative, indicating that the model generally overestimates the peak amplitude compared to observations. However, the differences are small in many cases, such as kors (−0.05 m) and hens (−0.05 m), suggesting a good match between observed and simulated first peak amplitudes. From figure 6, the first peak arrival times are generally close to the observed times. Stations like kwaj (6.6598) and hens (6.6598) have minimal discrepancies, indicating high accuracy in timing predictions. The statistical parameters shown in figure 6 further support the model’s reliability for arrival times, demonstrating an almost perfect match. The statistical metrics shown for amplitudes indicate a tendency for slight overestimation but generally accurate predictions. The electronic supplementary material, table S3, presents the performance metrics for model simulated tsunami heights at stations shown in figure 5. Overall, the simulations closely match the observations, particularly in predicting arrival times, with amplitudes that are nearly accurate but slightly overestimated in some instances. A better match between observed and simulated tsunami heights is reflected in positive regression coefficients, high significance percentages and low NRMSD, NMAD and acceptable NNSE values. However, there are few stations (dutc, xmas) where the model’s performance is weak, indicating a need for further refinement to improve the accuracy and reliability of the predictions at these locations.

### 5.2. Chile tsunami event 27 February 2010

**Figure 7.**
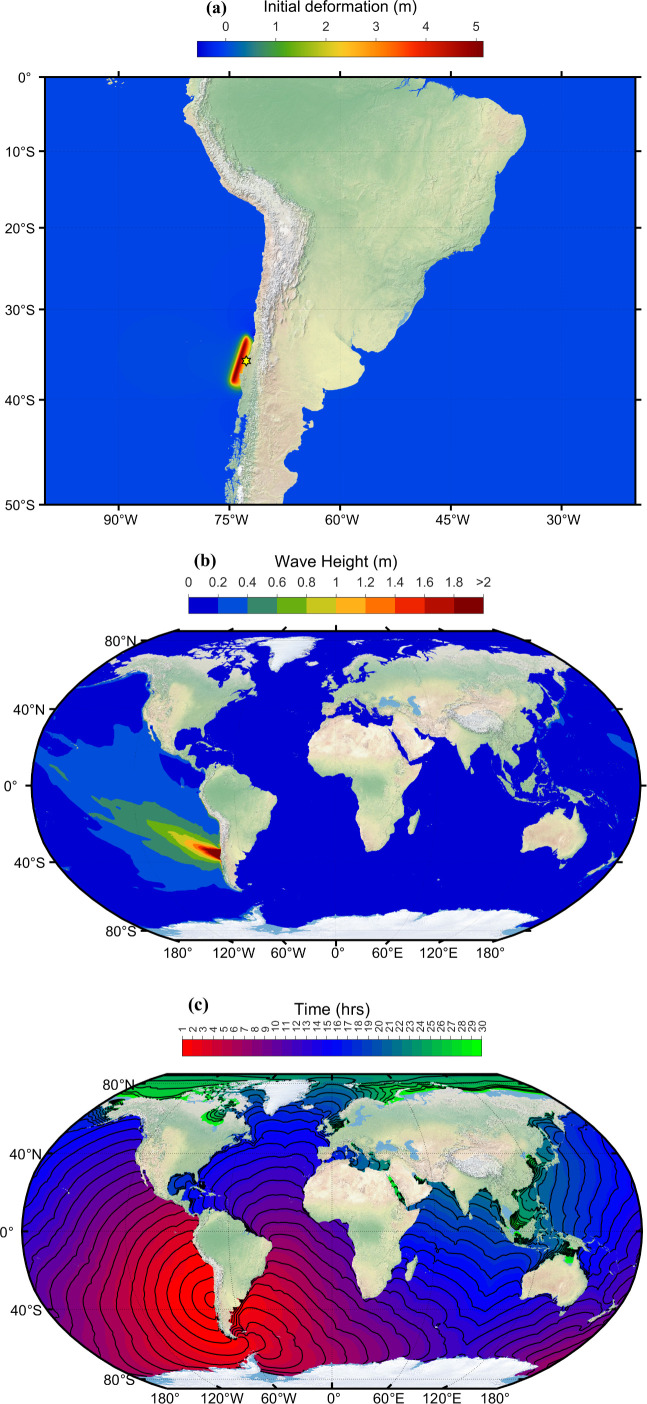
(*a*) Initial deformation of sea level. (*b*) Simulated maximum wave amplitude distribution. (*c*) Travel time contours of tsunami wave due to Chile tsunami event 27 February 2010. (The figure is on Robinson projection.)

**Figure 8.**
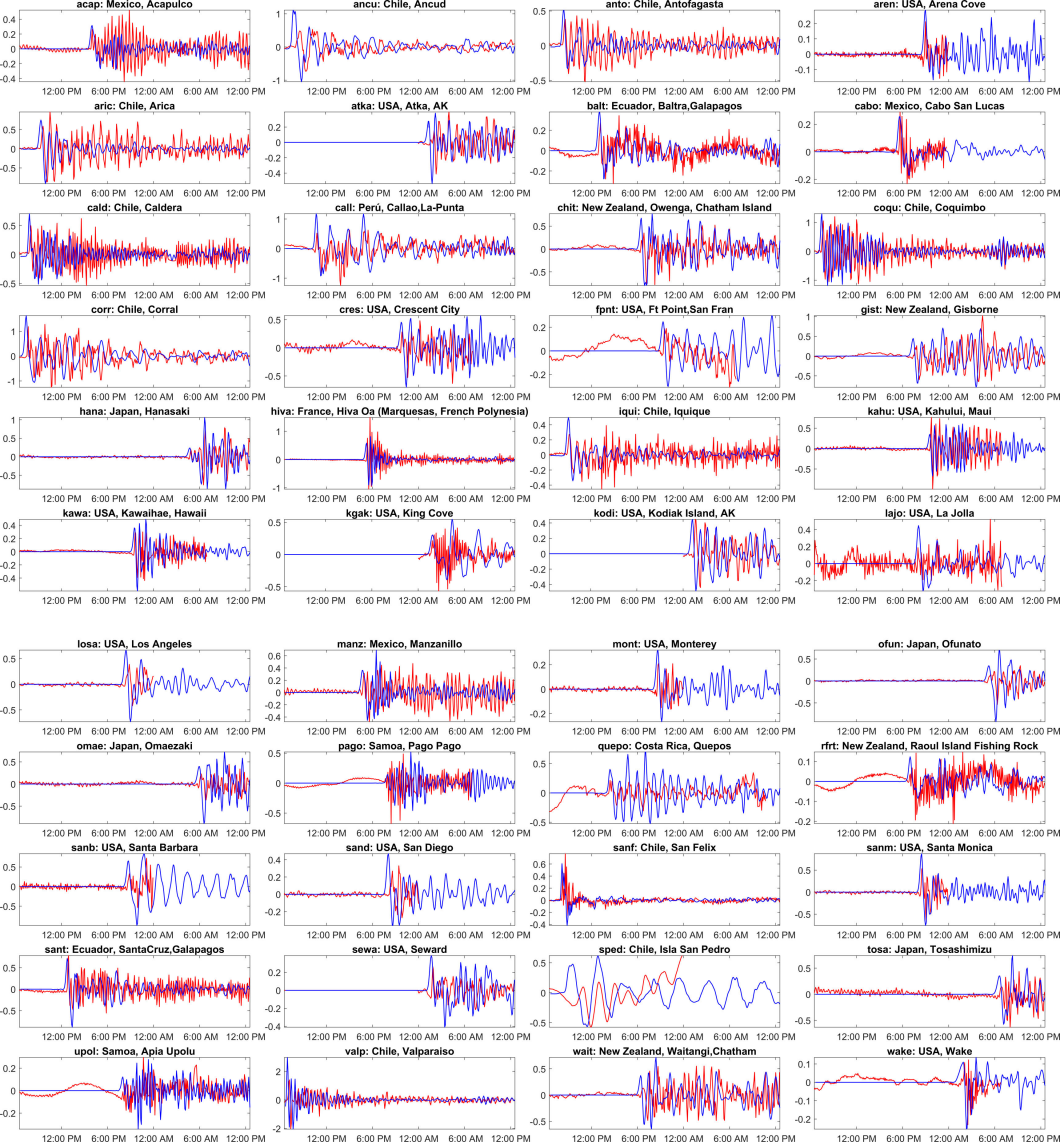
Comparison of the computed tsunami wave heights against the tide gauge observations at various global locations for the Chile 2010 event. The red colour corresponds to tide gauge observations, and the blue colour represents a numerical simulation. The locations of the tide gauge stations shown as titles on each subplot are marked in figure 3 and shown in table 1. The *x*-axis represents the time stamp, and the *y*-axis represents the wave height amplitude in metres. The starting time in the abscissa in each subplot is 27-Feb−2010 06:34:23 UTC. (See electronic supplementary material, table S4, for statistical performance metrics.)

On 27 February 2010, at 06:34:23 UTC (local time 03:34:23am), an earthquake of magnitude 8.9 Mw occurred approximately 325 km southwest of the Chilean capital of Santiago, off the coast of Chile’s Maule region [37]. The earthquake occurred under 37 km depth and was characterized by a thrust forcing mechanism caused by the Nazca Plate subducting beneath the South American Plate. This force displaced large volumes of seawater above and triggered a tsunami. A tsunami warning was issued first for Chile and Peru; Chile was significantly affected by a tsunami, with over 500 deaths, leaving 1000 s homeless and causing damage to properties and the economy [45]. The tsunami affected Chile’s coastline and generated warnings and evacuations across the Pacific Ocean, including as far away as Japan, New Zealand and the west coast of the United States. Figure 7*a* shows the initial deformation generated due to Chile’s 2010 earthquake. The focal fault parameters to generate initial deformation are shown in table 3. The distribution of peak tsunami wave height simulated using ADCIRC is shown in figure 7*b*. Travel time contours of tsunami waves at every 1 h interval are displayed in figure 7*c*. The ADCIRC-simulated water levels at different global locations are compared against the IOC sea level observations in figure 8. All these observations are taken from the IOC sea level website and are plotted in alphabetic order based on the station ID. The observations are averaged to five min from one min and displayed in figure 8. The tide gauge ID, country and location name are given as titles on each subplot, which can be accessed from table 1 for further information. From figure 8, the blue curves representing the ADCIRC simulations match the red curves representing the observations. The model did a good job of capturing the timing and first-wave amplitude of the tsunami waves. It can be observed from figure 8 that at most of the stations, the first peak tsunami height is slightly overestimated, and the wave arrival is a few minutes earlier. Although the first wave arrived and was captured at some locations, acap, anto, aric, manz, etc., the model underestimated the secondary tsunami wave heights. This is because, after the earthquake, there were multiple aftershocks as mentioned in the previous case, which would lead to tsunami waves, and these shocks are not considered in the current simulations. The model results can be further improved by employing exact bathymetry information and bottom friction information at the locations.

Figure 8 shows the widespread propagation of the 2010 Chile tsunami based on the different tsunami wave heights at different coastal countries in the Pacific Ocean. Chile faced the devastating blow, with waves greater than a metre high crashing into a few areas. Chile had waves ranging from 0.4 to 1.3 m in different cities like Ancud (figure 8; ancu), Coquimbo (figure 8; coqu) and Valparaíso (figure 8; valp). Other countries closer to Chile, like Peru and Ecuador, faced waves less than a metre, with Callao (figure 7; call), Peru, at 0.5 m and San Telmo (figure 8; sant), Ecuador, at 0.78 m. In North America, places like Costa Rica, Mexico and the USA had smaller waves, ranging from 0.3 to 0.6 m. While waves were generally smaller, San Diego (figure 8; sanb, sand) felt the highest peak at 0.6 m. Costa Rica’s Quepos (figure 8; quepo) recorded a 0.4 m peak, while Mexican waves ranged from 0.3 m in Acapulco (figure 8; acap) to 0.5 m in Manzanillo (figure 8; manz). The wave’s energy weakened but remained detectable further across the Pacific Ocean, away from North and South American continents and to other continents. In Oceania, Samoa and New Zealand experienced tsunami with waves between 0.2 and 0.5 m. Both Pago (figure 8; pago) and Upolu (figure 8; upol) in Samoa recorded peaks of 0.5 and 0.2 m, respectively. New Zealand’s Waitomo (figure 8; wait) saw a 0.5 m peak. In Asia, Japan’s coast saw a wave peak ranging from 0.4 to 0.7 m, with Hanasaki (figure 8; hana) experiencing the highest. Even far away in Europe, an island saw a wave of 0.6 m (figure 8; hiva).

**Figure 9.**
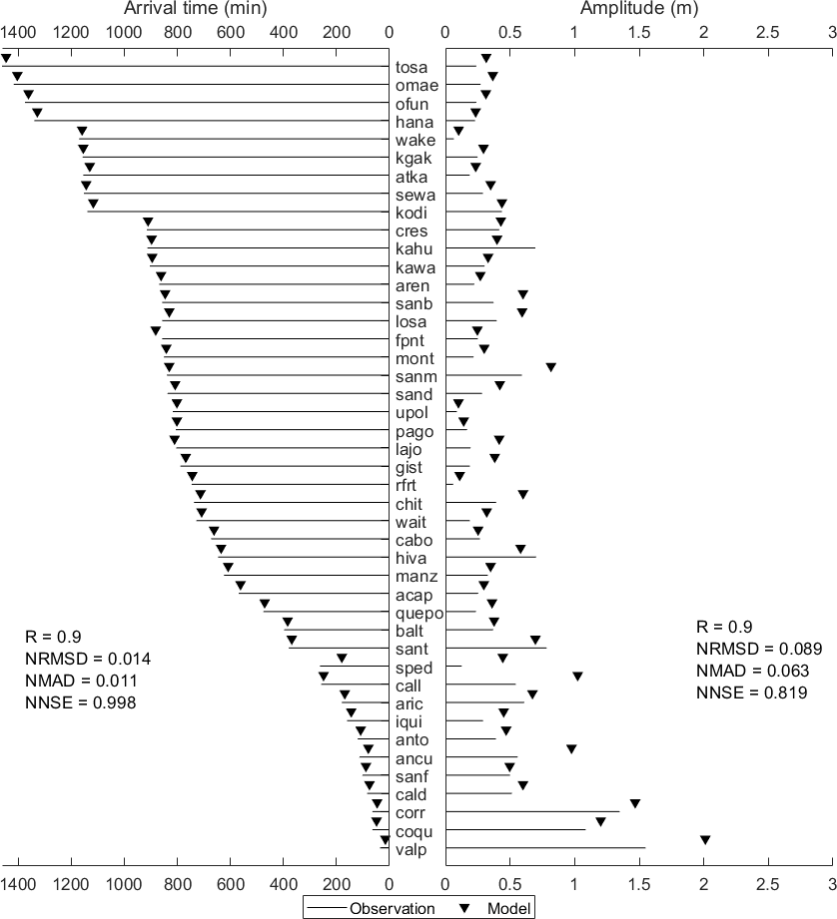
Comparison of observed and simulated first peak values for travel time and amplitude for the Chile tsunami event of 27 February 2010. The location of each station is indicated in figure 3, and the details are shown in table 1.

Figure 9 compares observed and simulated values for travel time and amplitude of the first tsunami peak at various stations (sorted by distance from the epicentre, with the closest station at the bottom) for the Chile tsunami event. Arrival times and amplitudes at multiple stations exhibit minimal differences between observed and simulated values with very good performance metrics. For most stations, the simulated tsunami wave arrived a few minutes earlier than observed (except for station fpnt). Additionally, the simulated amplitudes are consistently closer to or match the observations. Furthermore, statistical parameters displayed in figure 9 support the model’s reliability for both arrival times and amplitudes. These parameters demonstrate a good agreement between modelled and observed values (electronic supplementary material, table S4), providing a more detailed evaluation of the model’s performance at various stations (shown in figure 8) using statistical metrics for tsunami wave heights. Many stations show strong correlations and high significance levels. Additionally, high NNSE values with lower NRMSD and NMAD indicate accurate simulations. Overall, the model effectively captured the tsunami behaviour at all stations, demonstrating good performance across the domain with high significance and acceptable error metrics.

### 5.3. Indian ocean event 26 December 2004

**Figure 10.**
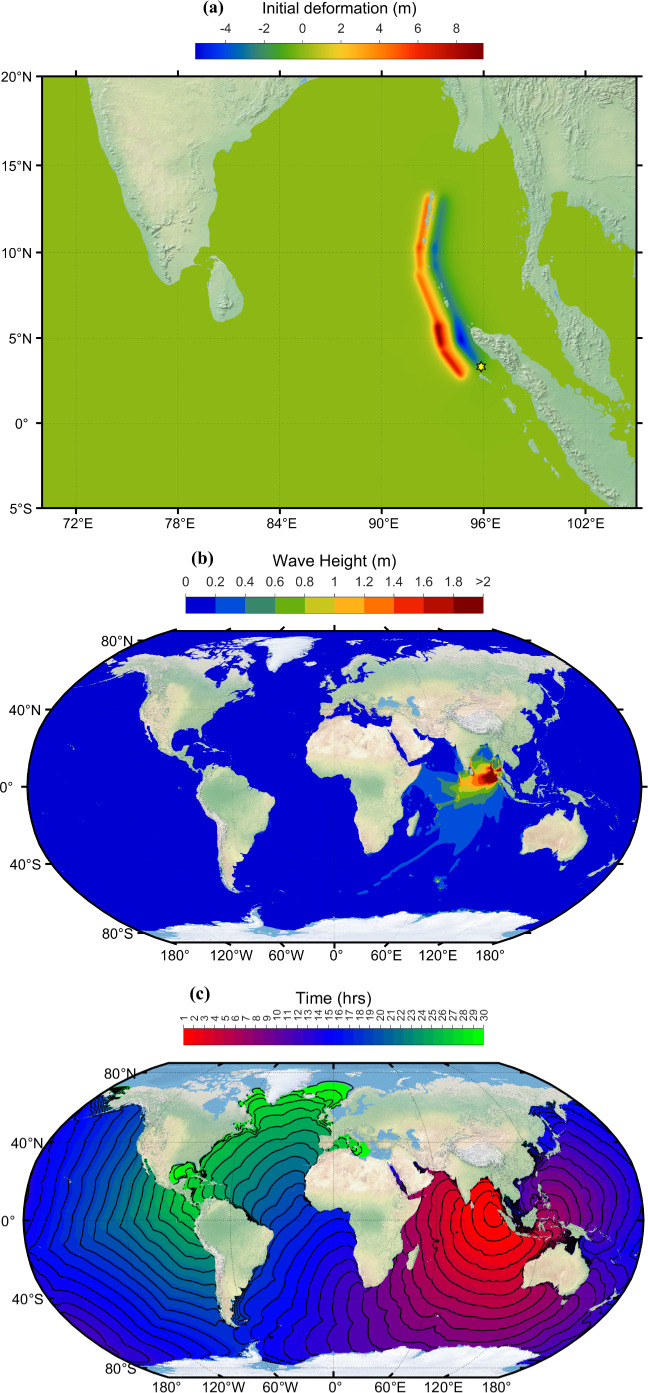
(*a*) Initial deformation of sea level. (*b*) Simulated maximum wave amplitude distribution. (*c*) Travel time contours of tsunami wave due to Indian Ocean tsunami event 26 December 2004. (The figure is on Robinson projection.)

The 2004 Indian Ocean tsunami, or the Boxing Day tsunami, is one of the deadliest natural disasters in recorded history. The tsunami was triggered by a massive undersea megathrust earthquake of 9.1−9.3 magnitude struck off the west coast of northern Sumatra, Indonesia and generated widespread destruction across countries bordering the Indian Ocean. Around 230 000 people died, thousands of people went missing, and millions of people were displaced from the coast [24]. In addition to human casualties, the impact was also felt in terms of economic losses and damage to infrastructure. Indonesia, Sri Lanka, India, Thailand and the Maldives were the most severely affected nations. The 2004 Indian Ocean tsunami highlighted the importance of disaster preparedness and international cooperation, leading to the development of new tsunami warning centres and enhanced tsunami early detection systems in the region, emphasizing the need for improved global collaboration to mitigate the impact of future catastrophic events [4]. Figure 10*a* shows the initial deformation generated due to the 26 December 2004 earthquake. The focal fault parameters are taken from Grilli *et al*. and are shown in table 3. The distribution of peak tsunami wave height simulated using current methodology is shown in figure 10*b*. Travel time contours of tsunami waves at every 1 h interval are displayed in figure 10*c*. Figure 10*b*,*c* is on Robinson’s projection. Figure 10*b* depicts that the countries India, Sri Lanka, Maldives, Indonesia and Thailand experience tsunami heights greater than 2 m. The tsunami wave took 2–3 h after the earthquake to reach Indian coasts.

**Figure 11.**
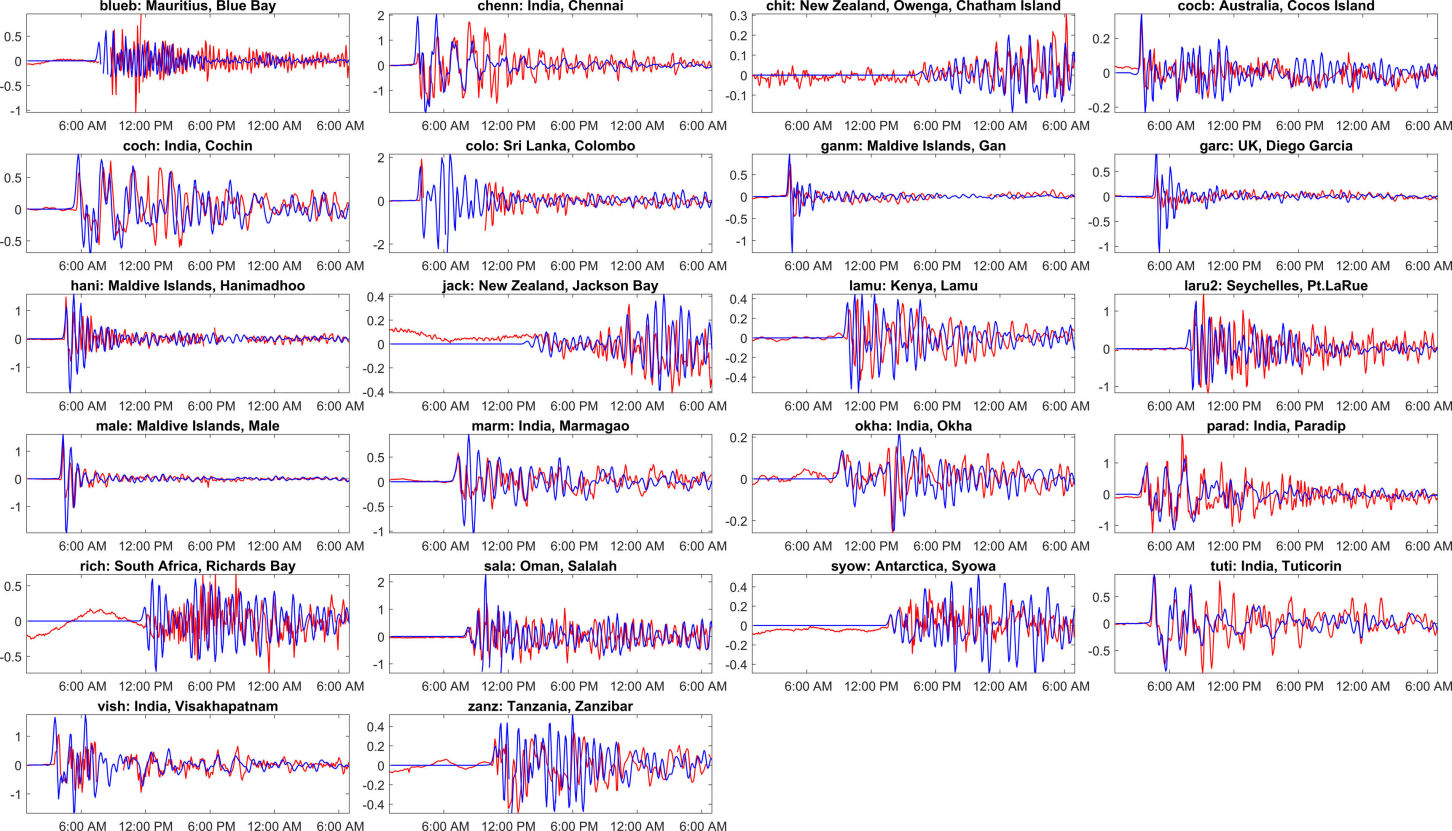
Comparison of the computed tsunami wave heights against the tide gauge observations at various global locations for the Indian Ocean 2004 event. The red colour corresponds to tide gauge observations, and the blue colour represents a numerical simulation. The locations of the tide gauge stations shown as titles on each subplot are marked in figure 3 and shown in table 1. The *x*-axis represents the time stamp, and the *y*-axis represents the wave height amplitude in metres. The starting time in the abscissa in each subplot is 26-Dec−2004 00:58:50 UTC. (See electronic supplementary material, table S5, for statistical performance metrics.)

**Figure 12.**
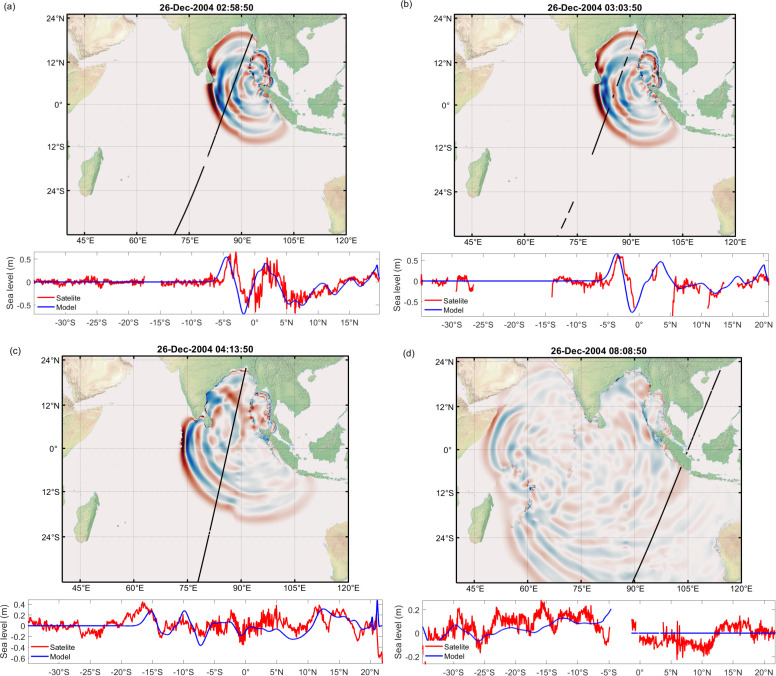
(*a*–*d*) Comparison between the sea height deviations as measured by the satellite altimeters and the modelled tsunami wave heights generated by the 26 December 2004, Sumatra earthquake (*a*) 2:58:50 hours, (*b*) 03:03:50 hours, (*c*) 04:13:50 hours and (*d*) 08:08:50 hours after the earthquake. These times coincide with the overflights of the satellites GFO, Jason-1, Envisat and TOPEX/Poseidon.

The simulated time series of water levels at different global locations are compared against the IOC sea level observations in figure 11. Red lines represent the observations, while blue lines show the model simulation in figure 11. Figure 11 conveys that the model is capturing the timing and amplitude of the tsunami waves well, which is evident from stations like Mauritius (blueb), Sri Lanka (colo) and the Maldives (ganm, male). There are some discrepancies between the model and observations at some locations; the model underestimates the wave height at Chennai, India (chenn) and overestimates at Lamu, Kenya (lamu). The model does not capture the smaller waves that arrived after the main tsunami at some locations (figure 11; blueb, chenn, tuti), whereas the same are visible at stations like Richards Bay, South Africa (rich) and Salalah, Oman (sala). The tsunami also reached Antarctica (figure 11; syow), which is captured in the current model and matches the observation well. Overall, the model provides a good representation of the 2004 tsunami. However, the results can be further improved by using the exact bathymetry and bottom friction coefficient. Figure 12 compares the ADCIRC computed wave heights against the satellite altimeters measured heights at different time stamps. The satellite altimetry data is downloaded from the link https://nctr.pmel.noaa.gov/indo_1204.html. The top plots in figure 12*a–d* illustrate the simulated propagation of the tsunami waves at 2.00 a.m., 2.05 a.m., 3.15 a.m. and 7.10 a.m. post the earthquake. These plots also show the satellite-traced path as a black line. The bottom plots show the comparison of wave heights along the path against the satellite altimetry measurement. It can be seen from the plots that ADCIRC is able to simulate the heights of the tsunami waves accurately and is a good match with the satellite observations.

**Figure 13.**
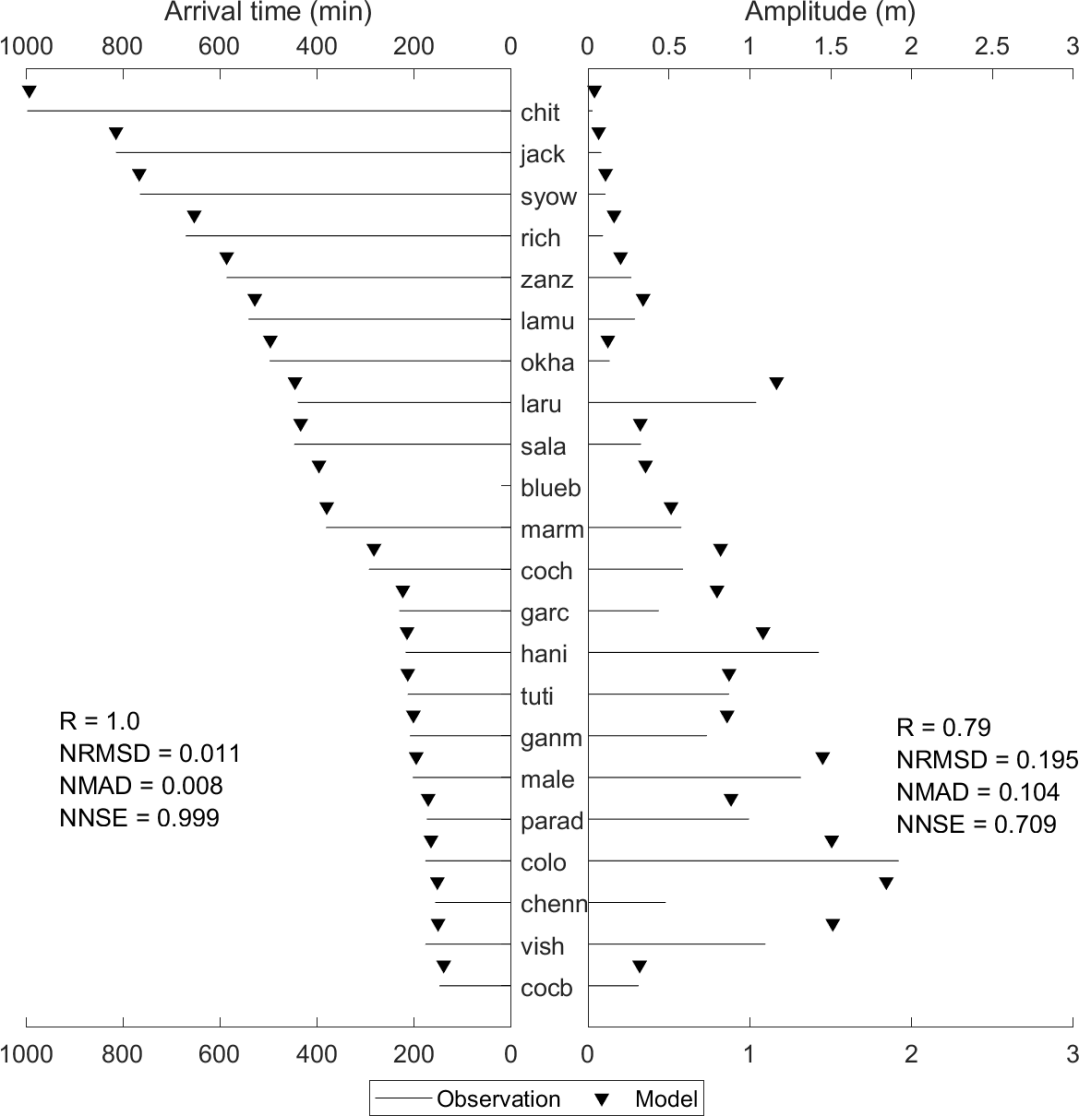
Comparison of observed and simulated first peak values for travel time and amplitude for the Indian Ocean tsunami event of 26 December 2004. The location of each station is indicated in figure 3, and the details are shown in table 1.

Figure 13 provides information on the first peak arrival time and amplitude between observed and simulated values for the 26 December 2004 tsunami event across various stations. The stations are sorted in ascending order (bottom to top) by distance from the epicentre. For the station ‘blueb’, the first peak amplitude is missing from the observations, and it is not shown in figure 13. For all the stations, the arrival times match well with the observations, showing good statistical performance metrics. For stations 'chenn' and 'vish' the amplitudes are overestimated by a large value. A close alignment between observations and simulations is indicated by smaller differences in both amplitude and arrival time, which is also evident by the statistical performance metrics displayed in figure 13. The electronic supplementary material, table S5, provides a comprehensive analysis of the model’s performance in predicting tsunami wave heights at various stations in the order shown in figure 11. Higher significance percentages across most of the stations indicate robust statistical reliability. Notably, most of the stations demonstrate excellent model performance with low error metrics and high efficiency. The stations jack, zanz show very poor regression but better NNSE, indicating the model can be improved at these stations. While the model performs well in many regions, certain stations with lower efficiency and higher deviation metrics highlight areas for further refinement and improvement.

### 5.4. Alaska tsunami event 28 March 1964

**Figure 14.**
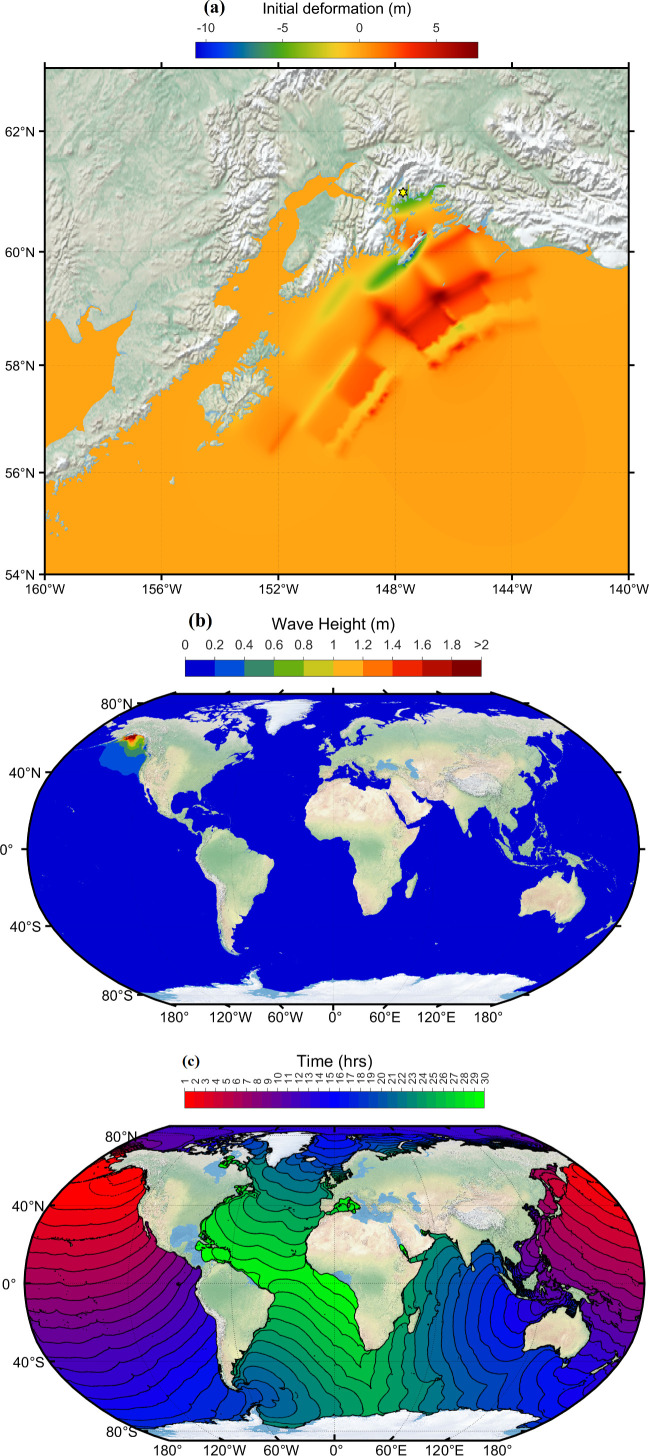
(*a*) Initial deformation of sea level. (*b*) Simulated maximum wave amplitude distribution. (*c*) Travel time contours of the tsunami wave due to the Alaska tsunami event 28 March 1964. (The figure is on Robinson projection.)

The 1964 Alaskan earthquake, also known as the Great Alaskan earthquake, Prince William Sound and Good Friday earthquake, occurred on 28 March 1960 at 03:36:00 UTC (Local time 27 [46] at 17:36:16). This massive earthquake is the most powerful earthquake recorded in North American history and the second-largest earthquake ever recorded globally. The earthquake was 9.2 Mw, and its epicentre was north of Alaska’s Prince William Sound region. The earthquake ruptured a large area beneath the continental margin of Alaska from Prince William Sound to Kodiak Island, triggering a tsunami. The tsunami devastated Alaska, and the earthquake also generated multiple local tsunamis due to submarine and subaerial landslides in coastal Alaska [47]. Figure 14*a* shows the initial deformation generated due to the 1964 Alaska earthquake. This deformation is obtained using a finite fault solution from [48]. A finite fault solution is a multi-fault representation of the rupture with variable local slip, rake angle, depth, rise time and rupture velocity. Figure 14*b*,*c* display the distribution of peak tsunami wave height simulated and travel time contours of tsunami waves at every 1 h interval for the current tsunami. Figure 14*b*,*c* is on Robinson’s projection. The deformation pattern shown in figure 14*a*, with subsidence of 10 m and uplift of 5 m, is a typical megathrust earthquake in a subduction zone. Figure 14*b* shows that the coastal locations of the Alaska region have experienced wave heights greater than 2 m.

**Figure 15.**
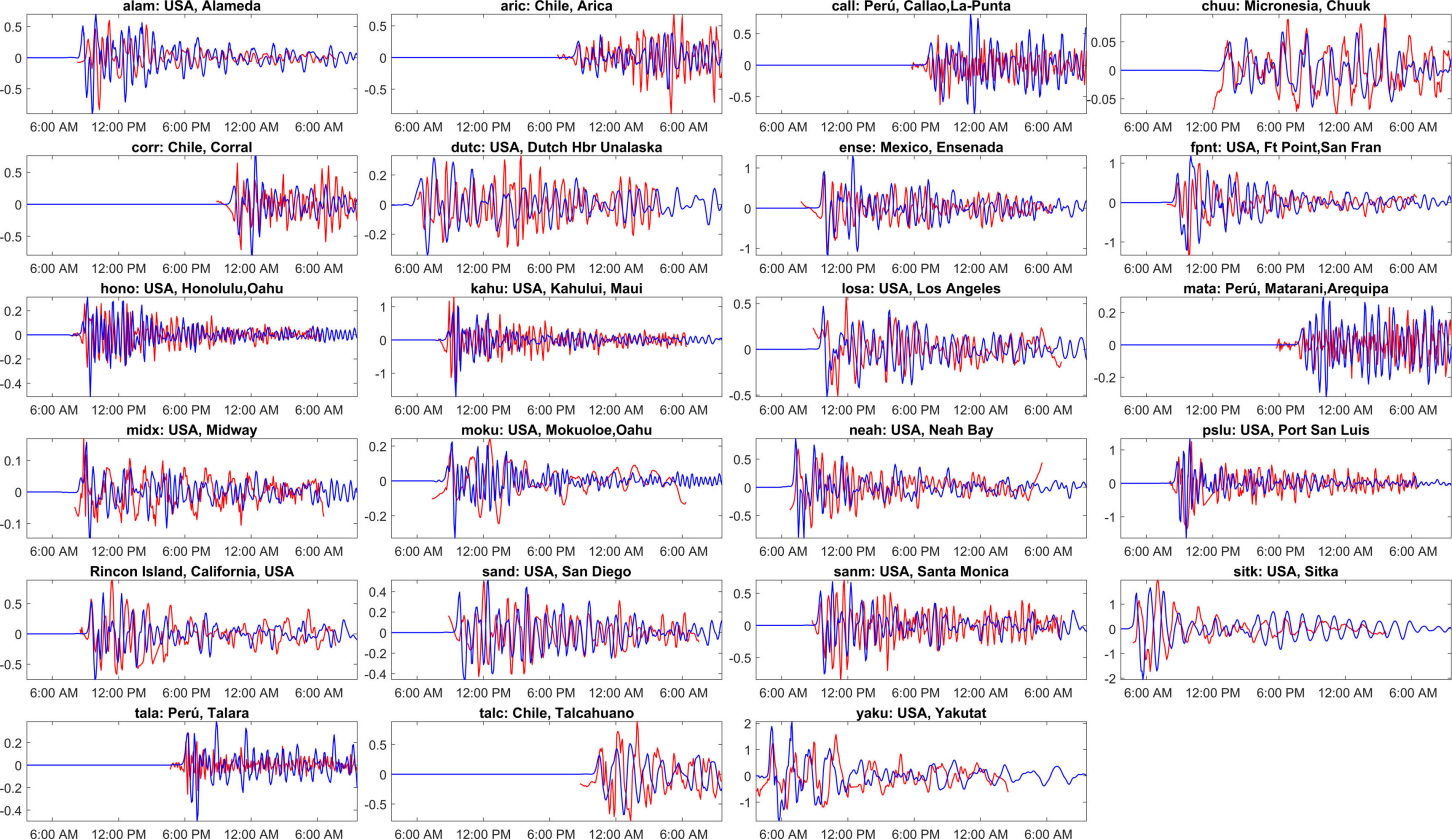
Comparison of the computed tsunami wave heights against the tide gauge observations at various global locations for the Alaska 1964 event. The red colour corresponds to tide gauge observations, and the blue colour represents a numerical simulation. The locations of the tide gauge stations shown as titles on each subplot are marked in figure 3 and shown in table 1. The x-axis represents the time stamp, and the y-axis represents the wave height amplitude in meters. The starting time in the abscissa in each subplot is 28-Mar−1964 03:36:00 UTC. (See electronic supplementary material, table S6, for statistical performance metrics.)

Figure 15 shows the time series of ADCIRC simulated water levels at different locations against the observations. The red and blue lines represent the observations and model simulations, respectively. The *x*-axis displays the time in hours after the earthquake’s origin time, and the *y*-axis shows the water level in metres relative to sea level. The titles on each subplot show the station code and location as per the IOC sea level website. The observations are taken at various tide stations along Alaska’s coastlines and other locations extending to South America and the Pacific Ocean to Hawaii. The observations used for the current tsunami are taken from [46]. Figure 15 shows that the model could capture the aspects of the tsunami, but there are slight discrepancies with the observations at a few locations. The model matches the arrival time of the first wave peak at most locations very well, but at a few locations, it slightly underestimates the water level. The model appears to capture the wave behaviour and overall trend against observation. Furthermore, the discrepancies in the model predictions completely depend on the initial deformation, bathymetry and the physical parameters used for the modelling. From figure 15, the highest recorded first peak arrival heights were observed at Yakutat, Alaska (figure 15; yaku), with a height of 1.3 m, followed closely by Sitka, Alaska (figure 15; sitk) at 1 m. These observations indicate the tsunami’s impact on coastal areas closer to the epicentre. Moving away from Alaska, the tsunami waves travelled southwestward, affecting coastal regions along the Pacific Ocean. As the waves propagated across the Pacific Ocean, their heights gradually reduced at locations farther from the earthquake’s epicentre. For instance, coastal locations in Washington state and California, such as Neah Bay (figure 15; neah), San Francisco and Los Angeles, recorded lower first peak arrival heights ranging from 0.3 to 0.6 m. Ensenada, Mexico (figure 15; ense), experienced a high first peak arrival height of 0.9 m, indicating the continued propagation of the tsunami across the Pacific Ocean. The tsunami reached Peru and Chile with varying arrival heights, ranging from 0.2 to 0.6 m at locations such as Talara (figure 15; tala), La Punta (figure 15; call), Arica (figure 15; aric) and Talcahuano (figure 15; talc). The tsunami waves travelled further across the Pacific Ocean, reaching the Hawaiian Islands. Honolulu (figure 15; hono), Kahului (figure 15; kahu) and other Hawaiian locations (figure 15; moku, midx) recorded arrival heights ranging from 0.2 to 1 m, indicating the impact of the tsunami on these distant islands.

**Figure 16.**
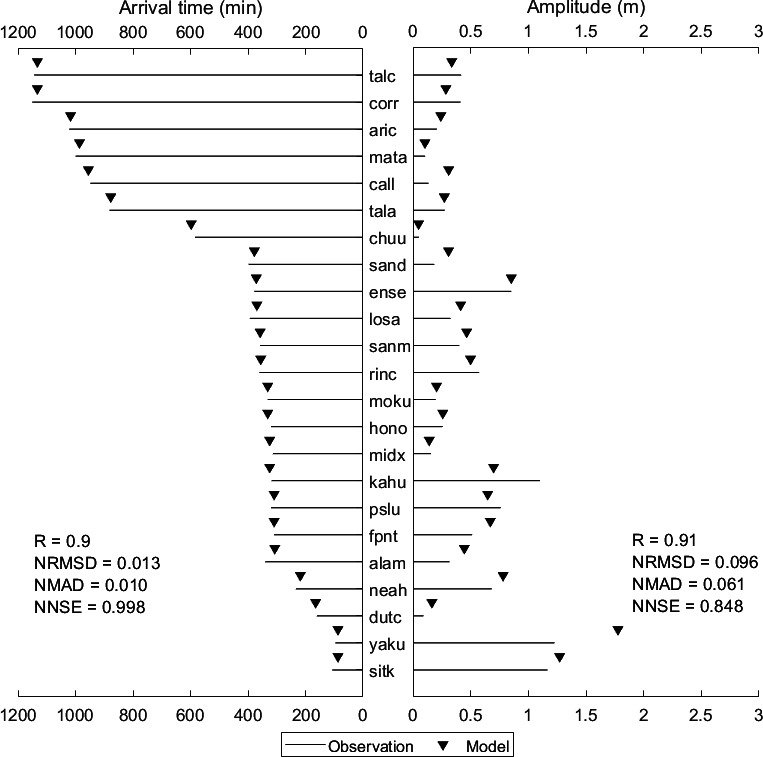
Comparison of observed and simulated first peak values for travel time and amplitude for the Alaska tsunami event of 28 March 1964. The location of each station is indicated in figure 3, and the details are shown in table 1.

Figure 16 shows the observed and simulated values for the first peak arrival time and amplitude at various stations during the Alaska 1964 tsunami event. The stations are arranged in ascending order of distance from the epicentre (top to bottom). The majority of stations have perfect matches to small differences in arrival times and amplitudes. Stations like 'yaku' and 'alam' exhibit differences in both arrival times and amplitudes, highlighting the areas needing improvement. From figure 16 and looking at the statistical performance metrics displayed, overall, the model performed well but requires further refinement for consistent accuracy across all the stations. The electronic supplementary material, table S6, presents various statistical metrics to evaluate the performance of ADCIRC-modelled tsunami wave height across different stations, as shown in figure 15. Regression analysis shows high significance percentages at many stations, suggesting a strong statistical reliability in the results. The NRMSD and NMAD values are relatively low, implying a decent match between observed and simulated wave heights. The NNSE values are mostly above 0.3 (except at stations call, mata), with several stations exceeding 0.4, reflecting good predictive accuracy and model performance. Overall, the model demonstrates competent performance in predicting tsunami wave heights but shows variability in accuracy across different stations.

### 5.5. Valdivia tsunami event 22 May 1960

**Figure 17.**
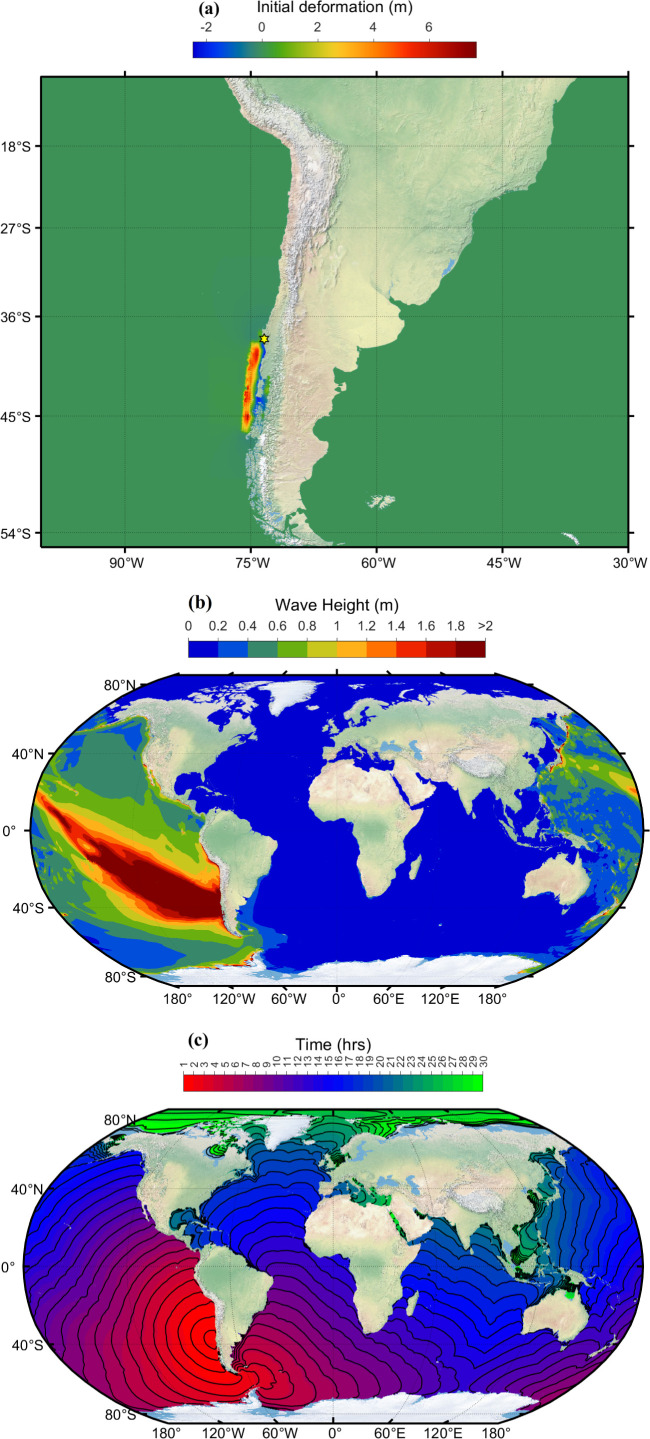
(*a*) Initial deformation of sea level due to earthquake. (*b*) Simulated maximum wave amplitude distribution. (*c*) Travel time contours of tsunami wave due to Valdivia tsunami event 22 May 1960. (The figure is on Robinson projection.)

The 1960 Valdivia earthquake, also known as the Great Chilean Earthquake, occurred on 22 May 2006 at 19:11:20 UTC (local time 22 May 1960 15:11:14) near Valdivia in Southern Chile. The earthquake had a magnitude of 9.5, making it the most powerful earthquake ever recorded. This megathrust earthquake triggered a massive tsunami that affected the coastal countries across the Pacific Ocean. The tsunami waves reached as far as Hawaii, Japan, the Philippines, New Zealand and even to the west coast of the United States. The destruction caused by this earthquake and tsunami significantly impacted scientific developments and understanding. The data provided by the earthquake was valuable and helped scientists understand plate tectonics, prompting advancements in seismological research and monitoring. Figure 17*a* shows the initial deformation generated due to the 1960 Valdivia earthquake. This deformation is obtained by employing a finite fault solution. The finite fault solution used to generate the initial deformation is taken from [38]. A finite fault solution is a multi-fault representation of the rupture with variable local slip, rake angle, depth, rise time and rupture velocity. The distribution of peak tsunami wave height simulated for this tsunami is shown in figure 17*b*. Travel time contours of tsunami waves at every 1 h interval are displayed in figure 17*c*. Figure 17*b*,*c* is on Robinson’s projection. It can be seen from figure 17*b* that the coastal countries of South America and a few parts of North America have experienced tsunami heights greater than 2 m. Tsunami waves reached coastal parts of Japan 12 h after the earthquake, and even parts of Japan experienced wave heights greater than 1 m.

**Figure 18.**
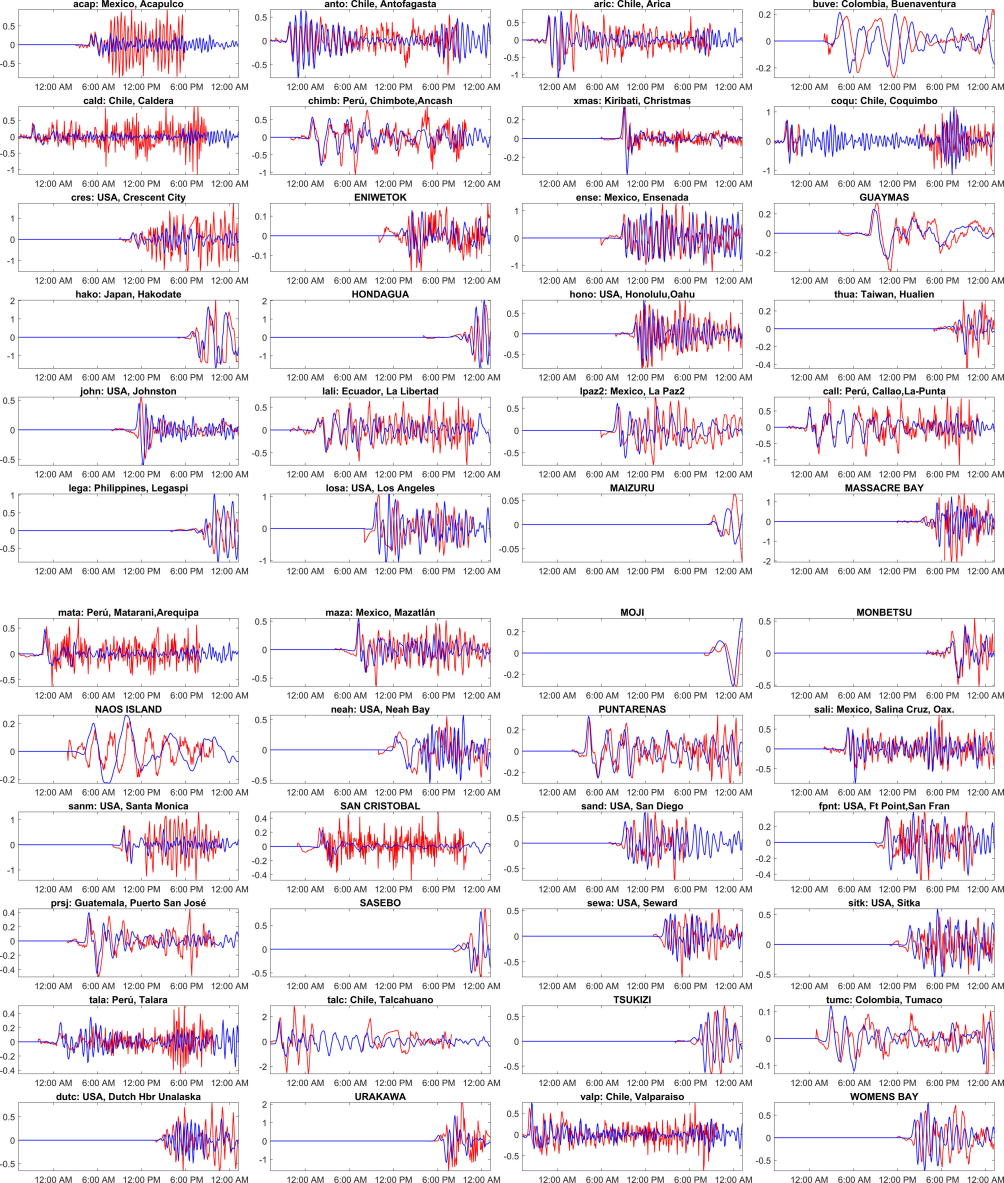
Comparison of the computed tsunami wave heights against the tide gauge observations at various global locations for the Valdivia 1960 event. The red colour corresponds to tide gauge observations, and the blue colour represents a numerical simulation. The locations of the tide gauge stations shown as titles on each subplot are marked in figure 3 and shown in table 1. The *x*-axis represents the time stamp, and the *y*-axis represents the wave height amplitude in meters. The starting time in the abscissa in each subplot is 22 May 1960 19:11:20 UTC. (See electronic supplementary material, table S7, for statistical performance metrics.)

Figure 18 compares the simulated time series of water levels at different global locations against the sea level observations. The red and blue lines represent the observations and model simulations in figure 18. The observations for this tsunami event are downloaded from [38]. From figure 18, it can be observed that the simulated results match the observations well. At most locations, the first peak wave is captured close to the observation in amplitude and time. In the previous case events, there was a slight mismatch in arrival times and an overestimated peak at a few locations. This could be because of the generation mechanism of initial deformation [41]. In contrast to previous cases, the current simulation used a finite fault solution for the generation as the data are available; this incorporates variable slip across the fault plane, whereas in the previous simulations, a single fault parameter was used. The discrepancies due to the uncertainties in deformation values using uniform slip distribution can be avoided by using finite fault solution. Furthermore, tsunami waves arrived at a few locations after the first peak (figure 18; acap, cald, cres, sanm, etc.), but the model underestimated this. This could be due to aftershocks not being considered in the current simulations. Also, this might be due to the time dependencies in dynamic rupture along the fault, which is not considered in the current simulations. The tsunami travelled great distances across the Pacific Ocean and affected several countries. The earthquake’s epicentre was off the coast of Chile, and the country experienced devastating effects along the coastal regions (figure 18; talc, valp, coqu, anto, cald, aric). The tsunami had an impact in Peru (figure 18; mata, call, chimb, tala), USA (figure 18; cres, hono, john, losa, neah,sanm, sand, fpnt, sitk, dutc) and Mexico (figure 14; acap, ense, lpaz2, maza, sali). The tsunami reached far Japan (figure 18; Monbetsu, Urakawa, hako, Tsuziki, Maizuru, Moji, Sasebo), Taiwan (figure 18; thua) and the Philippines (figure 18; Hondagua, ega) causing destruction and few casualties.

**Figure 19.**
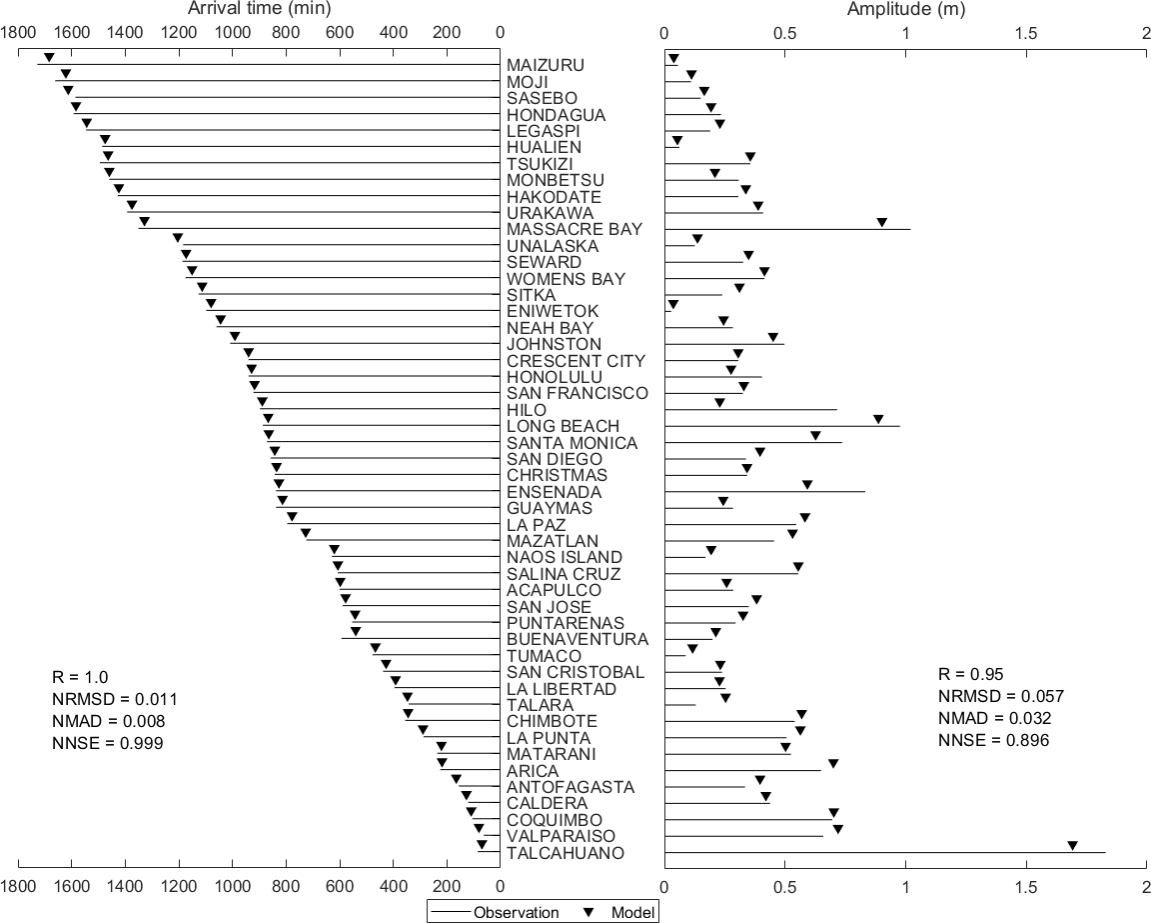
Comparison of observed and simulated first peak values for travel time and amplitude for the Valdivia tsunami event on 22 May 1960. The location of each station is indicated in figure 3, and the details are shown in table 1.

Figure 19 shows observed and simulated values of the first peak arrival time and the first peak amplitude along with statistical performance metrics across various stations for the Valdivia tsunami. The stations are arranged from bottom to top in ascending order w.r.t. distance from the epicentre. From figure 19, it can be conveyed that the model shows good performance at most of the stations, with arrival times close to the observations with minimal amplitude differences. For a few stations, VALPARAISO, UNALASKA and SASEBO, the amplitudes are close to the observations, but arrival time is delayed compared to the observations, indicating the model needs some refinement at these locations towards the arrival times. The electronic supplementary material, table S7, presents statistical metrics assessing the performance of a tsunami model at various stations, as shown in figure 18. The R and SS% the model’s reliability, with many stations achieving 100%, highlighting statistical significance between observed and modelled wave heights. From electronic supplementary material, table S7, the NRMSD and NMAD values show that the average discrepancies between observed and simulated values are lower, denoting better simulations. For many stations, the higher NNSE values indicate a better predictive performance of the model. Overall, the model demonstrates good performance at several stations, highlighting the model’s capability to capture tsunamis.

## 6. Conclusion

ADCIRC’s ability to solve SWE efficiently stems from its message passing interface-based parallel processing capabilities and its FE approach. This enables swift simulations, critical for operational tsunami warning centres (TWC) to quickly disseminate vital information to emergency responders, authorities and the public, facilitating timely evacuations and reducing potential casualties. ADCIRC’s real-time tsunami simulation capability, simulating tsunami propagation across global or regional scales within minutes, is invaluable for providing timely forecasts of coastal impacts.

Regarding computational requirements, ADCIRC benefits greatly from HPC environments, where real-time applications typically use several hundred to a few thousand processing cores, depending on the scale and resolution needed. However, dependency on HPC infrastructure can pose challenges, especially for TWC in resource-limited regions. While ADCIRC excels in simulating deep water wave propagation, its computational demands increase in nearshore areas where higher resolution is needed to capture finer coastal details. Mesh refinement, essential for accurately modelling coastal impacts, can significantly increase computational time, particularly when very high resolution is required. To mitigate this, operational centres can apply finer mesh resolution only in critical coastal areas or regions of interest while maintaining coarser resolution in deeper waters. Additionally, pre-running scenarios for frequently occurring seismic sources can reduce real-time computational demands by allowing simulations to start from pre-calculated initial conditions, further enhancing operational efficiency. The accuracy of wave heights and arrival times largely depends on bathymetry and real-time estimates of the earthquake’s magnitude, geographic extent and the slip on the fault. Using high-resolution bathymetry and accurate seismic fault parameters can significantly improve model accuracy.

The current study demonstrates the successful application of the FE-based ADCIRC model for global tsunami simulations, mainly focusing on distant tsunamis and assessing their threat to Indian coasts. Through simulations of major historical tsunamis, such as the 2011 Japan, 2010 Chile, 2004 Indian Ocean, 1964 Alaska and 1960 Valdivia events, the research establishes ADCIRC’s reliability in predicting tsunami wave heights and arrival times. Comparisons with tide gauge observations from various global locations show a close alignment between model predictions and observed data, with favourable statistical performance metrics confirming the model’s accuracy. While slight overestimations of peak wave heights and early arrival times were observed, this can be advantageous for operational purposes, as it allows for earlier evacuations. The global unstructured mesh, with spatial resolutions ranging from 2 km in shallow waters to 20 km in deeper regions, effectively balances computational efficiency and simulation accuracy. The real-time simulation capabilities of ADCIRC open avenues for enhanced global tsunami hazard modelling and risk mapping, offering critical insights for TWC in identifying coastal hot spots and issuing timely warnings to mitigate potential damages and save lives.

However, based on the findings in the study and the observed discrepancies between the model and tide gauge data, there is scope for further improvement, which will be addressed in future work. One area of focus will be the inclusion of astronomical tides in the computations, as local tides can significantly influence tsunami wave behaviour. Additionally, aftershocks will be incorporated into the model, enhancing its ability to account for secondary seismic events. Another important aspect is the inclusion of local wind waves, particularly for regions like the Indian coasts, where high waves during monsoons may contribute to the amplification of tsunami waves. Furthermore, the use of spatially varying Manning coefficients and bottom friction data across the domain will improve model accuracy in simulating coastal impacts. These advancements will contribute to more precise and operationally efficient tsunami predictions.

## Data Availability

The official release version of ADCIRC is available from the project website at http://adcirc.org/. The ADCIRC code can also be found in the GitHub repository: [49]. The bathymetry data used for meshing in the current study can be downloaded from https://download.gebco.net/. The OceanMesh2D MATLAB package used for meshing can be accessed from: [50]. The observations and model data used for plotting time series for five tsunami events are attached as a supplementary file in Excel format [51].
